# NFAT1 and NFAT3 Cooperate with HDAC4 during Regulation of Alternative Splicing of PMCA Isoforms in PC12 Cells

**DOI:** 10.1371/journal.pone.0099118

**Published:** 2014-06-06

**Authors:** Michalina Kosiorek, Paulina Podszywalow-Bartnicka, Ludmila Zylinska, Slawomir Pikula

**Affiliations:** 1 Department of Biochemistry, Nencki Institute of Experimental Biology, Warsaw, Poland; 2 Department of Neurodegenerative Disorders, Laboratory of Neurogenetics, Mossakowski Medical Research Centre PAS, Warsaw, Poland; 3 Laboratory of Cytometry, Nencki Institute of Experimental Biology, Warsaw, Poland; 4 Department of Molecular Neurochemistry, Medical University, Lodz, Poland; Univ. Kentucky, United States of America

## Abstract

**Background:**

The bulk of human genes undergo alternative splicing (AS) upon response to physiological stimuli. AS is a great source of protein diversity and biological processes and is associated with the development of many diseases. Pheochromocytoma is a neuroendocrine tumor, characterized by an excessive Ca^2+^-dependent secretion of catecholamines. This underlines the importance of balanced control of calcium transport via regulation of gene expression pattern, including different calcium transport systems, such as plasma membrane Ca^2+^-ATPases (PMCAs), abundantly expressed in pheochromocytoma chromaffin cells (PC12 cells). PMCAs are encoded by four genes (*Atp2b1*, *Atp2b2*, *Atp2b3, Atp2b4),* whose transcript products undergo alternative splicing giving almost 30 variants.

**Results:**

In this scientific report, we propose a novel mechanism of regulation of PMCA alternative splicing in PC12 cells through cooperation of the nuclear factor of activated T-cells (NFAT) and histone deacetylases (HDACs). Luciferase assays showed increased activity of NFAT in PC12 cells, which was associated with altered expression of PMCA. RT-PCR experiments suggested that inhibition of the transcriptional activity of NFAT might result in the rearrangement of PMCA splicing variants in PC12 cells. NFAT inhibition led to dominant expression of 2x/c, 3x/a and 4x/a PMCA variants, while in untreated cells the 2w,z/b, 3z,x/b,c,e,f, and 4x/b variants were found as well. Furthermore, chromatin immunoprecipitation experiments showed that NFAT1-HDAC4 or NFAT3-HDAC4 complexes might be involved in regulation of PMCA2x splicing variant generation.

**Conclusions:**

We suggest that the influence of NFAT/HDAC on PMCA isoform composition might be important for altered dopamine secretion by PC12 cells.

## Introduction

Alternative splicing of pre-mRNA is a major post-transcriptional source of protein diversity, which is essential for a variety of biological processes, both under physiological and pathological conditions [1]. Recent genome-wide association studies have shown that 94% of human multi-exon genes undergo alternative splicing [2]. In the nervous system, alternative splicing is switched on and off during different processes, including learning, memory, synaptogenesis or neurotransmission, by modulation of neurotransmitter release, ion channel functions, and receptor specificity [3]–[5]. In the nervous system, alternative splicing of genes encoding the neural cell adhesion molecule (NCAM), NMDA receptors, and calcium pumps, for example the plasma membrane Ca^2+^-ATPase (PMCA), undergoes cell activity-induced changes [6]–[10]. Instabilities in alternative splicing regulatory sequences and disturbances in the binding of regulatory proteins to these sequences are important causes of numerous human diseases [11]. This is especially true for neurodegenerative diseases, neurological tumors and mental disorders [12], [13]. One of the commonly known neuropathologies is the pheochromocytoma neuroendocrine tumor, which causes widespread consequences such as hypertension or cardiac arrhythmia, as well as psychiatric disturbances [14]. Pheochromocytoma is localized in the adrenal medulla and is characterized by an excessive secretion of catecholamines, i.e. epinephrine, norepinephrine and dopamine.

Pheochromocytoma chromaffin cells (PC12 cells) release neurotransmitters in the process of Ca^2+^-regulated exocytosis [15]. Thus, PC12 cells are equipped with the neuronal type of secretory machinery, demanding tight Ca^2+^-dependent genetic control over alternative splicing of mRNAs encoding proteins involved in the maintenance of calcium homeostasis and secretory response [16]. Accordingly, alterative splicing has been found to influence the expression profile of various mRNAs encoding secretory proteins [17], including elements of membrane fusion complex: SNAP25, syntaxin 1 and synaptobrevin 1 [18]–[21] and mRNAs encoding calcium transporters (calcium pumps, ions exchangers, calcium channels). A great number of examples have been given on the alternative splicing of mRNAs for voltage gated calcium channels [22], [23], sodium calcium exchangers [24], [25], and plasma membrane Ca^2+^-ATPases (PMCAs). The latter proteins are the main subject of the several important studies [8], [26]. PMCAs are responsible for pumping Ca^2+^ ions out of the cell and maintenance of low cytosolic calcium ions concentration ([Ca^2+^]_c_). PMCAs are encoded by four genes (*Atp2b1*, *Atp2b2*, *Atp2b3, Atp2b4)* and some exons of these genes might be excluded from or included into the final mRNA/transcript by the process of alternative splicing generating almost 30 mRNA transcript variants [8], [10]. PC12 cells express all PMCA isoforms, and most of the splicing variants [26]. Alternative splicing of PMCAs affects two strategic regions of the pump: the acidic phospholipid-binding domain (splice site A) and the Ca^2+^-calmodulin binding domain (splice site C) [8]. Thus, alternative splicing generates PMCA variants of different structure and biochemical properties, such as affinity for calcium ions, velocity of calcium ion transport or ability to interact with a different signaling proteins (e.g. calcineurin, nitric oxide synthase, calmodulin or 14-3-3 protein) [9], [27]–[29]. Expression profile of the alternatively spliced variants of PMCAs has been well established in various tissues [8], [27], [30], [31]. However, the molecular basis of generation of alternative transcripts of PMCAs, including molecular mechanisms and regulatory proteins that may induce or arrest this process, remains unclear. Alternative splicing has been recently described as a co-transcriptional process requiring activity of transcription factors, histone modifying proteins and other regulatory proteins involved in chromatin rearrangement [32]–[39]. Transcriptional factors may influence alternative splicing by interaction with RNA polymerase II, which is responsible for targeting of the splicing machinery to the site of transcription [40]. One of the transcription factors whose activity has been linked with alternative splicing is nuclear factor of activated T cells (NFAT). NFAT was found to influence the alternative splicing of mRNAs of allograft inflammatory factor-1 (AIF-1) [41], of interferon responsive transcript-1 (IRT-1) [20], and of synaptotagmin-like 2 protein [42]. Interestingly, NFAT has been proposed to be responsible for the control of the expression of the PMCA1 and PMCA4 isoforms [43]–[48]. As already mentioned, histone modification could be another important tool for the control and moderation of the alternative splicing process. Several histone-binding proteins were found to interact with splicing factors [49]–[53]. Among the proteins that modify histones, histone deacetylases (HDACs) play an extremely important role, both in the context of the regulation of gene expression, by influencing the availability of DNA as well as in the context of alternative splicing of mRNA [54]. Furthermore, HDACs were found to interact with NFATs and to repress their activity [55]. More precisely, the class IIa of HDACs have been shown to repress cardiac hypertrophy by inhibiting cardiac-specific transcription factors such as myocyte enhancer factor 2 (MEF2), GATA4, and NFAT in the heart [56]. On the other hand, it was shown that NFATc1 favored the binding of HDAC3 to the proximal region of the osteocalcin gene promoter, enhancing the expression of the gene [57]. Finally, our recent studies have suggested that overactive NFAT signaling is responsible for the repression of genes *Vamp1* and *Vamp2* in PC12 cells, stressing the importance of NFAT activity in these cell types [58]. The interdependence between HDAC and NFAT suggests that these proteins could counteract or cooperate during the regulation of alternative splicing of mRNAs of PMCAs. In this report we would like to propose putative regulatory mechanisms of Ca^2+^-dependent alternative splicing of plasma membrane Ca^2+^-ATPases (PMCAs), that might be switched on during excessive secretion of catecholamines in pheochromocytoma. Thus, this study may shed a new light on the role of the genetic diversity of PMCA in a number of different neuropathologies as well as during cognitive processes based on neurotransmission.

## Materials and Methods

### Cell Culture and Cell Lines

PC12 cell line were obtained from the Department of Molecular Neurochemistry, Medical University of Lodz (purchased from American Type Culture Collection, ATCC No.: CRL-1722). PC12 cells were cultured in RPMI-1640 medium (Sigma Aldrich, USA) with 10% heat-inactivated horse serum and 5% heat-inactivated fetal bovine serum (Gibco, Invitrogen), in an atmosphere of 5% CO_2_/95% air at 37°C. In some experiments the inhibitor of NFAT (1 µM 11R-VIVIT) (Calbiochem Merck Chemicals, Germany) was added for 48 h to the cell culture. Cell lines deficient in PMCA2 (_2) or PMCA3 (_3) were established as described previously [59], [60]. Control cells (C) were transfected with empty pcDNA3.1 (+) plasmid. Stable cell lines were obtained by a 4-week selection with 1 mg/ml G418 and used for the experiments at passages 15–30. Using flow cytometry, cells were routinely tested propidium iodide staining in terms of cell cycle and apoptosis, and none of used conditions (5 mM KCl, 59 mM KCl or 1 µM 11R-VIVIT) did affected the measured parameters.

### Quantitative PCR

Total RNA was isolated using TRIzol Reagent (Invitrogen). RNA aliquots of 5 µg were subjected to reverse transcription using MuMLV reverse transcriptase (Promega, USA), according to the manufacturer’s protocol. The obtained cDNA (3 µl) was used in quantitative PCR (qPCR) with the SYBR Green reagent (Applied Biosystem) according to the manufacturer’s protocol. qPCR data were normalized to *Gapdh* and *18SrRNA* expression and calculated according to the ΔΔC_T_ method [61]. The calculations for 11R-VIVIT treated cells were carried out according to a modified ΔΔC_T_ method as follows: ΔΔC_T_ = ΔC_11R-VIVIT-treated_−ΔC_non-treated_. PMCA isoform expression was verified by RT-PCR. The primers were designed for the *R. norvegicus* genome using the GenScript Primer Design Tool (USA) (Table 1) and used at 1 µM concentration.

**Table 1:** Primers for PMCA isoforms expression analysis.

gene name	sequence (5′ → 3′)	strand	Tm °C	amplicon size(bp)	NCBI number
*Atp2b1*	AGAAGTTCACCGTCATCAGG	F	57.71	92	NM_053311.1
	ATCACCGTACTTCACTTGGG	R	57.51		
*Atp2b2*	TTGCTGTCAGGAACTCATGT	F	56.76	79	NM_012508.5
	TGCCAGTTTGAGAGTTGACA	R	57.95		
*Atp2b3*	GAAAGCAGGATTGGTGATGT	F	57.58	164	NM_133288.1
	CAACCAACACAGTGACTCCA	R	58.06		
*Atp2b4a*	GAAATCCAGCCACTCAACAG	F	58.29	148	NM_001005871.1
	ACATGATCAGACCTGCCTTC	R	57.64		
*Atp2b4b*	GAATAACAATGCCGTGGACT	F	57.54	189	NM_001005871.1
	GGAGCAGCTGATGAAACAAT	R	57.89		

### RT-PCR for Alternative Splicing of PMCAs

To estimate the alternative splicing of PMCA isoforms the RT-PCR was performed. RNA aliquots of 15 µg or 10 µg were subjected to reverse transcription (RT) as described above. The obtained cDNA (10 µl) was used to assess PMCA alternative splicing at site A and at site C. PCR was performed using recombinant *Taq* DNA polymerase (Invitrogen, USA) and 2 µM primers (Table 2) designed as described [62]. The PCR product bands were visualized by electrophoresis in 1.5% agarose gel with 0.5 µg/ml ethidium bromide by UV transillumination.

**Table 2:** Primers for PMCA alternative splicing analysis.

gene(accession number)	sequence (5′−3′)	Tm (°C)	strand	splice site	splice variant	PCR product(bp)
*Atpb2b1* *(*NM_053311.1)	CTTACCTTACTTGGAGCTG	55.00	F	A	w	504
	GTTGTTATCCTTCATCATTTTCTT	57.00	R			
	ATCTTCTGCACAATTGTCTTAG	57.00	F	C	b	358
	GAGCTACGAATGCATTCACC	58.00	R			
*Atpb2b2*(NM_012508.5)	CTGTGGGTGTCAACTCTCAA	58.00	F	A	w, y, x, z	348, 306, 255, 213
	GTGAGCTTGCCCTGAAGCA	59.00	R			
	CATCTTCTGCACCATCGTTC	58.00	F	C	b, a, c	417, 644, 472
	AGCCATGAAGTTATGGATGGA	57.00	R			
*Atpb2b3*(NM_133288.1)	CATGTCATGGAAGGTTCTGG	58.00	F	A	y, x, z	200, 521, 479
	GTTATTGTCCTTCATCATTTTCTT	57.00	R			
	ATCTTCTGTACCATTGTCCTG	57.00	F	C	b, a, c, e, g	203, 357, 290, 444, 424
	GAGCTACGGAATGCTTTCAC	58.00	R			
*Atpb2b4*(NM_001005871.1)	GTGACTGCTGTGGGAATCAA	58.00	F	A	x, z	524, 488
	GTTGTTGTCCTTCATCATTTTCTT	57.00	R			
	TCTGCTCTGTTGTTTAGGCA	56.00	F	C	b, a	343, 518
	ATGAAATACTTTGACCACTCTG	57.00	R			

### Total Cell Lysate Preparation and Immunoblotting

Cells were harvested and washed with PBS at room temperature. Cells were incubated for 40 min in an ice-cold lysis buffer (10 mM Tris-HCl, pH 7.5, 80 mM NaCl, 1% Triton X-100, 1 mM dithiothreitol, 1 mM PMSF, 10 mM NaF, 2 mM Na_3_VO_4_ and 10 µg/ml protein inhibitor cocktail, Sigma Aldrich). The lysates were centrifuged at 800×g for 5 min at 4°C and stored at −20°C. The protein content was determined by the Bradford assay. Total cell lysates were analyzed by SDS-PAGE and immunoblotting as described [60] using the antibodies described in table 2 (Table 2 and Table S1).

### Luciferase Reporter Assay

Luciferase reporter plasmids with NFAT-dependent promoter (pGL3-NFAT-luc), *Renilla* luciferase control plasmids (pRL-SV40), promoter-less plasmid pGL3-luc and plasmid overexpressing NFAT (pNFAT+/+) were gifts from Dr. Wieslawa Lesniak from the Nencki Institute of Experimental Biology. PC12 cells (2×10^5^) were transfected with X-tremeGENE Transfections Reagent (Roche Applied Science, Germany) with the following plasmidcombination: pGL3-NFAT-luc with pRL-SV40, pGL3-luc with pRL-SV40 (negative control), pNFAT+/+, with pGL3-NFAT-luc and with pRL-SV40 (positive control). Cells were harvested 48 h after transfection and lysed in lysis buffer (Thermo Scientific Pierce). Firefly and *Renila* luciferase activities were assayed with Pierce *Renilla*-Firefly Luciferase Dual Assay Kit (Thermo Scientific Pierce). The luminescent signal from *Renilla* luciferase was measured at λ_max_ = 535 nm and that from firefly luciferase at λ_max_ = 613 nm. The working solution contained substrates for both luciferases (coelenterazine and D-luciferin), and the reactions occurred simultaneously with flash-type kinetics. The luminescent signals were spectrally resolvable using filters. The activity of NFAT was determined based on the luminescence signal from firefly luciferase and standardized to the signal from *Renilla* luciferase. The luminescence emission was determined by SpectraMax M5e Microplate Reader (Molecular Devices, Sunnyvale, California, United States). The efficiency of transfection was verified by transfections with plasmids overexpressing EGFP and assessed by means of fluorescence microscopy to be 20%.

### Immunoprecipitation

Cells were lysed in RIPA buffer and samples for immunoprecipitation were obtained with agarose beads with protein A/G (Santa Cruz, USA) as described [19]. Samples were incubated for 2 h at 4°C with mouse monoclonal anti-NFAT1 antibody (Abcam, USA) or with rabbit polyclonal anti-NFAT3 antibody (Cell Signaling, USA). The obtained immunoprecipitates were subjected to SDS-PAGE and immunoblotting with the following antibodies: mouse IgG1 anti-HDAC1 (10E2), mouse IgG1 anti-HDAC2 (3F3), mouse IgG1 anti-HDAC3 (7G6C5), rabbit polyclonal anti-HDAC4 (D15C3), rabbit polyclonal anti-HDAC5, rabbit polyclonal anti-HDAC6 (D2E5) (Cell Signaling, USA).

### Chromatin Immunoprecipitation (ChIP)

PC12 cells (2×10^7^) were cross-linked with 0.5% formaldehyde for 10 min at room temperature. Cross-linking was stopped by adding 125 mM glycine on ice. Cells were solubilized in a buffer containing 10 mM Tris-HCl (pH 8.0), 1% Triton X-100, 1% sodium deoxycholate, 1 mM PMSF and PIC for 10 min at 4°C. Pellets obtained by centrifugation at 1000×g for 5 min were suspended in RIPA buffer and sonicated using a Bioruptor Sonicator (Diagenode, Belgium) to shear chromatin into 500 bp fragments. Sonicated chromatin was subjected to immunoprecipitation using ChIP-grade agarose beads with protein G (Cell Signaling), blocked with 1% bovine albumin and 1% salmon sperm DNA. Then anti-NFAT1 and anti-NFAT3 antibodies were added and the obtained DNA-protein complexes were further complexed with anti-HDAC4 antibody (Cell Signaling). The obtained protein-DNA complexes were eluted with 100 mM sodium acetate and 1% SDS for 30 min and treated with RNase for 6 h at 65°C and proteinase K o/n at 45°C. DNA was isolated using the phenol/chloroform/isoamyl reagent (Sigma Aldrich, USA) and subjected to RT-PCR with primers used for analysis of the alternative splicing pattern of PMCA isoforms.

### Statistical Analysis

All data are presented as means ± SEM of n observations. All data were analyzed by the two-sample paired Student’s t test at 95% or 99% confidence. Two-way ANOVA test was used for internal comparison between all experimental groups (3 cell lines) in terms of treatment with 1 µM 11R-VIVIT. For qPCR the nonparametric paired Wilcoxon signed rank test was used at 95% or 99% confidence.

## Results

### Altered Composition of PMCA Isoforms Influence Calcium Homeostasis

The goal of our study was to present changes exerted by stable suppression of PMCA2 or PMCA3 isoform. Thus, we used an in vitro cellular model with permanently downregulated PMCA2 and PMCA3 expression, which was validated in our several other studies [58]–[60]. At this point a comment should be added why the RNAi method was not appropriate regarding assumptions of this research. RNAi involves short-lived molecules and induces transient changes, thus the decay rate of any observed changes may vary considerably. In case of stable transfection we could control the level of PMCA isoforms and monitor long term effects of their suppression.

We have already shown that experimental reduction in the PMCA2 or PMCA3 content in PC12 cells caused a significant drop in the efficiency of calcium removal [59]. This data suggested that the control of [Ca^2+^]_c_ in PC12 cells, especially the calcium clearance, was tightly dependent on the composition of PMCA isoforms, especially PMCA2 and PMCA3 isoforms, exhibiting the highest Ca^2+^ transport velocity and the highest affinity for Ca^2+^ [29]. The expression profile of PMCAs depends on a fine-tuned regulation by Ca^2+^-dependent molecular tools such as transcription factors or alternative splicing factors. Experimental downregulation of PMCA2 or PMCA3 created specific local calcium environment which was apparently important for the expression patter of the remained isoforms. Since we have observed an activation of Ca^2+^/calcineurin-dependent transcription factor NFAT, in the next stage of studies we have examined whether NFAT could be involved in determination of PMCA composition.

### Overactivation of NFAT in the Cells with Reduced Content of PMCA2 or PMCA3

**Figure 1:**
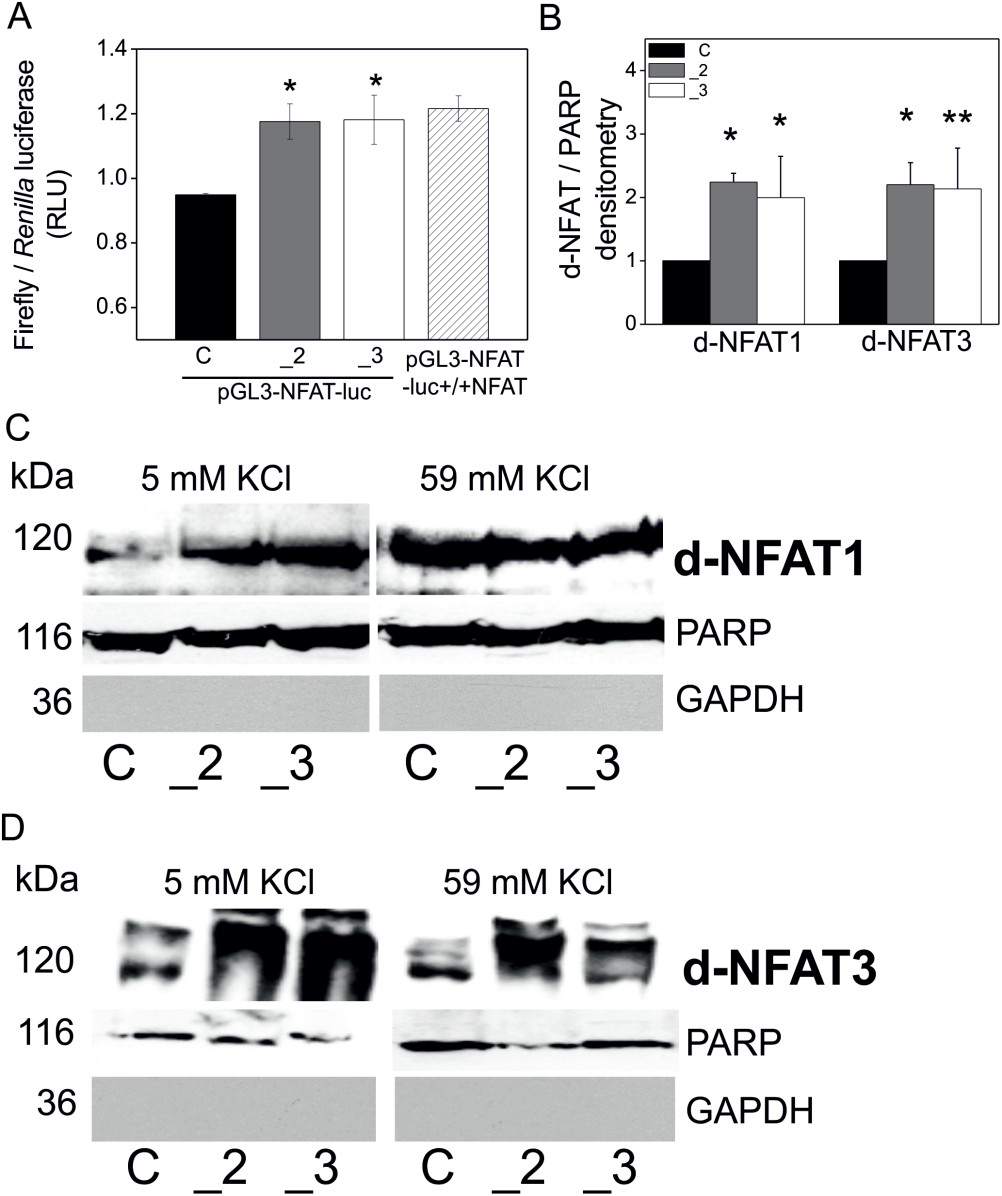
NFAT activation in PC12 cells with reduce PMCAs content. PC12 cells were transfected with plasmids encoding firefly luciferase under NFAT-dependent promoter (pGL3-NFAT-luc) and reference plasmids with *Renilla* luciferase (pRL-SV40). Negative controls were wild type PC12 cells transfected with promoterless pGL3-luc plasmids and positive controls were wild type PC12 transfected with plasmids overexpressing NFAT together with the pGL3-NFAT-luc (pGL3-NFAT-luc-NFAT+/+). NFAT activity was determined with a luciferase reporter dual assay (Thermo Scientific Pierce) and showed as the ratio of the luminescence signals derived from Firefly and *Renilla* luciferases. Bars represent mean values ± SEM. Symbols: control cells (C), PMCA2-deficient cells (_2), PMCA3-deficient cells (_3) and pGL3-NFAT-luc+/+NFAT – wild type cells overexpressing NFAT. Student’s t-test was used for comparison of control cells with PMCA2- or PMCA3-deficient cells. *P≤0.05, n = 5 (**A**). Nuclear content of dephosphorylated NFAT1 and NFAT3 was analyzed by immunoblotting. Protein bands were quantified densitometrically, standardized to PARP (nuclear marker) and normalized to control cells, expressed as y = 1. Bars represent mean values ± SEM. Student’s t-test was used for comparison of control cells with PMCA2- or PMCA3-deficient cells. *P≤0.05, n = 6 (**B**). Representative immunoblots of nuclear content of dephosphorylated NFAT1 (**C**) and NFAT3 (**D**) are demonstrated. Symbols correspond to: control cells (C), PMCA2-deficient cells (_2), PMCA3-deficient cells (_3).

Luciferase reporter assays performed using constructs containing NFAT-dependent promoter revealed that NFAT transcriptional activity was significantly higher in the cells with a reduced content of PMCA2 or PMCA3. Moreover, this increase in NFAT activation was comparable to the cells overexpressing NFAT (transfected with constructs pGL3-NFAT-luc-+/+NFAT) (Fig. 1A). In addition, a statistically significant increase in the protein content of dephosphorylated NFAT1 and NFAT3 in the nuclei has been detected in PMCA2- and PMCA3-deficient cells, especially under resting conditions. This was demonstrated by densitometrical measurements of the immunoblots of NFAT1 and NFAT3, standardized to the content of nuclear poly (ADP-ribose) polymerase (PARP) and normalized to control cells (y = 1) (Fig. 1B). Increased level of dephosphorylated NFAT1 and NFAT3 in the nuclei in PMCA-deficient cells under resting conditions and in all cell types upon plasma membrane depolarization was shown by representative immunoblots (Fig. 1C and Fig. 1D). These findings concerning NFAT activity reinforce the hypothesis on the existence of a NFAT-PMCA regulatory loop.

### PMCA Isoforms Expression Pattern upon Altered Transcriptional Control by NFAT

**Figure 2:**
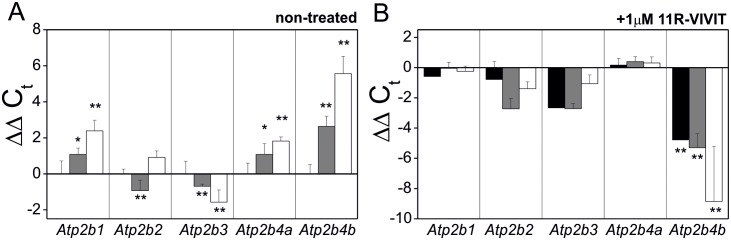
PMCA isoforms expression pattern versus transcriptional control by NFAT in PC12 cells. Expression of genes encoding PMCA isoforms: *Atp21b1* (PMCA1), *Atp21b2* (PMCA2), *Atp21b3* (PMCA3), *Atp21b4a* (PMCA4a), *Atp21b4b* (PMCA4b) was determined by qPCR for PC12 cells non-treated (**A**), and treated with NFAT inhibitor (1 µM 11R-VIVIT) (**B**). Bars represent mean values ± SEM. Wilcoxon test was used for ΔC_t_ from qPCR data (n = 3) for comparison between control cells (standardized to y = 0) and PMCA2- or PMCA3-deficient cells non-treated with 11R-VIVIT (n = 3). Two-way ANOVA test was used for comparison between non-treated and 11R-VIVIT treated cells. *P≤0.05, **P≤0.01.

Following the above, the expression level of genes encoding PMCA isoforms: *Atp21b1* (PMCA1), *Atp21b2* (PMCA2), *Atp21b3* (PMCA3), *Atp21b4a* (PMCA4a), *Atp21b4b* (PMCA4b) was determined by qPCR for PC12 cells non-treated (Fig. 2A), and compared with the cells treated with NFAT inhibitor (1 µM 11R-VIVIT) (Fig. 2B). On one hand, these experiments confirmed downregulation of PMCA2 or PMCA3 in respective cell lines. On the other hand, these experiments showed that inhibition of NFAT influenced significantly the expression pattern of PMCA4b as well as PMCA2 and PMCA3.

### Alternative Splicing of PMCAs Depends on Activity of NFAT in Splicing Regulatory Regions

**Figure 3:**
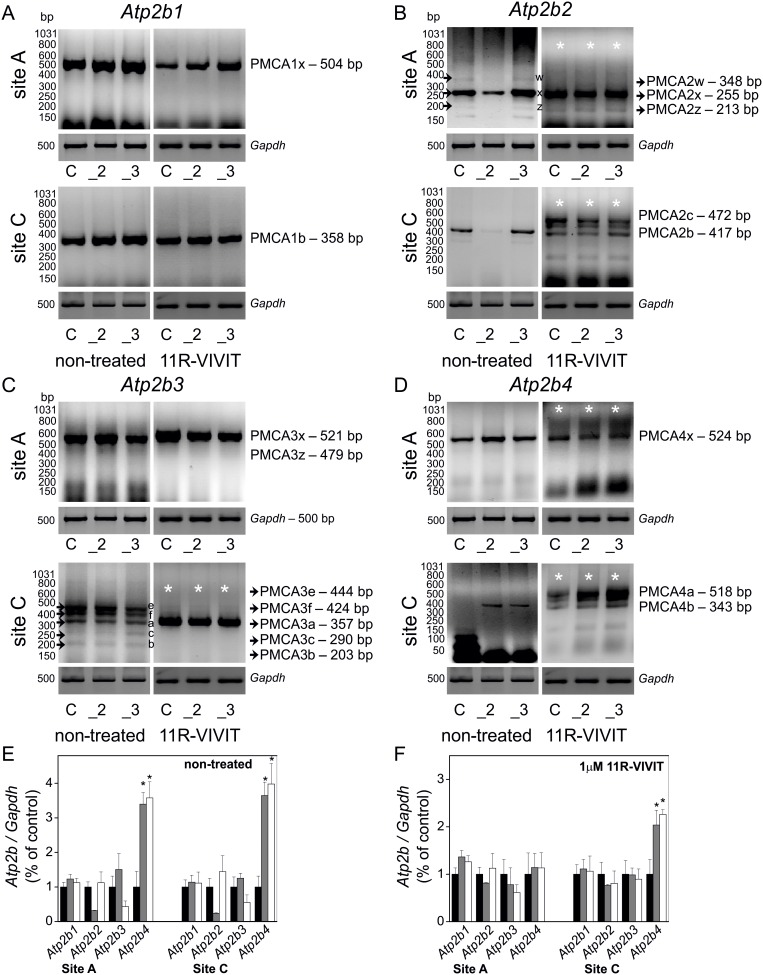
Alternative splicing of PMCA in PMCA2- or PMCA3-deficient PC12 cells upon NFAT inhibition. Alternative splicing pattern at sites A and C of mRNA transcripts of PMCAs: *Atp21b1* (PMCA1) (**A**), *Atp21b2* (PMCA2) (**B**), *Atp21b3* (PMCA3) (**C**), *Atp21b4* (PMCA4) (**D**) was determined by RT-PCR in non-treated and 1 µM 11R-VIVIT-treated PC12 cells. RT-PCR product bands were quantified densitometrically, standardized to *Gapdh* and normalized to control cells, expressed as y = 1, both for non-treated (**E**) and 11R-VIVIT-treated cells (**F**). Student’s t-test was used for comparison of control cells with PMCA2- or PMCA3-reduced cells (n = 3). Bars represent mean values ± SEM. *P≤0.05. Symbols: control cells (C), PMCA2-deficient cells (_2), PMCA3-deficient cells (_3). Black arrows indicate the PCR product bands for PMCA2 site A and PMCA3 site C. White asterisks on the images of gels indicate the PCR product bands generated by alternative splicing that underwent a significant change upon NFAT inhibition with 11R-VIVIT.

Diversity of PMCAs is not only due to the fact that these calcium pumps are encoded by four separate genes but mostly due to alternative splicing of mRNA. Thus, following the above findings suggesting increased NFAT activity and contribution to PMCAs expression profiling, a detailed analysis of the alternative splicing pattern of PMCA transcripts was performed. In order to obtain full information on the composition of PMCA splice variants the PCR method with primers flanking the appropriate splicing sites was applied. PMCA splicing variant composition was tested in control cells and in cells with a reduced content of neurospecific isoforms PMCA2 or PMCA3. This study was performed in order to verify whether a reduction in the content of neurospecific PMCAs might be compensated by other PMCA splicing variants. It revealed that downregulation of PMCA2 or PMCA3 did not statistically significantly influence the expression of PMCA1 (*Atp2b1*), and that the PMCA1x/b variant was abundantly and predominately expressed in PC12 cells (Fig. 3A, left). These outcomes were in accordance with the known data on PC12 cells [62], [63]. The expression of *Atp2b1* transcript was in agreement with the protein level of PMCA1, detected with a specific antibody recognizing the PMCA1b form (Fig. S1A). In the case of *Atp2b2* expression pattern, the PMCA2x/b variant predominated in all cell lines and its level was elevated in a compensatory mechanism in cells with a reduced amount of PMCA3 (Fig. 3B, left). This finding was verified at a protein level with a specific antibody recognizing the PMCA2b form (Fig. S1A). Regarding the expression pattern of *Atp2b3*, the most abundant variant was PMCA3x/a and in cells with a reduced amount of PMCA2 a compensatory increase in PMCA3x/a level was observed (Fig. 3C, left). PMCA3x/a transcript expression also correlated with the content of a protein recognized by specific anti-PMCA3a antibody (Fig. S1A). PMCA4x/b expression increased significantly in both PMCA2- and PMCA3-deficient cells (Fig. 3D, left). This was confirmed at a protein level by (Fig. S1A).

Putative contribution of NFAT to the generation of PMCA splice variants was tested in PC12 cells incubated in the presence of NFAT inhibitor, 1 µM 11R-VIVIT for 48 hours. Upon this treatment the expression level of the PMCA1x/b variant did not significantly alter (Fig. 3A, right). In the case of PMCA2 splicing pattern, NFAT inhibition led to predominant expression of PMCA2x/c and PMCA2x/b, while the PMCA2z/b and PMCA2w/b variants, as well as PMCA2z/c and PMCA2w/c, were almost unchanged (Fig. 3B, right). Regarding the profile of PMCA3 splicing, a brain-specific variant PMCA3x/a predominated in all cell lines upon NFAT inhibition, while the expression of other splicing forms at site A (PMCA3e,f,c,b) was completely abolished (Fig. 3C, right). Finally, NFAT inhibition led to predominant expression of PMCA4x/a over the PMCA4x/b variant, which is a brain-specific variant exhibiting higher affinity for Ca^2+^ ions and better efficiency in Ca^2+^ removal (Fig. 3D, right). All RT-PCR data on alternative splicing pattern of PMCAs were quantified densitometrically. RT-PCR product bands were measure densitometrically, standardized to *Gapdh* and normalized to control cells, expressed as y = 1, both for non-treated (Fig. 3E) and 11R-VIVIT-treated cells (Fig. 3F). Taking into account the above results, it is very likely that the activity of NFAT is necessary during alternative splicing of PMCA. In particular, NFAT might be involved in the formation of PMCA2w,z, and PMCA3e,f,c,b splice variants. Bioinformatic analysis of the spliced regions in introns and UTR of genes coding for PMCAs revealed the presence of target motifs for NFAT (5′-TTTCCC-3′, and 5′-GGGAAA-3′). Based on the bioinformatic analysis and distribution of these motifs, it can be assumed that NFAT might bind to the regulatory splicing sequences alone or in complexes with other regulatory proteins.

### Interaction of NFATs with HDACs in PC12 Cells

**Figure 4:**
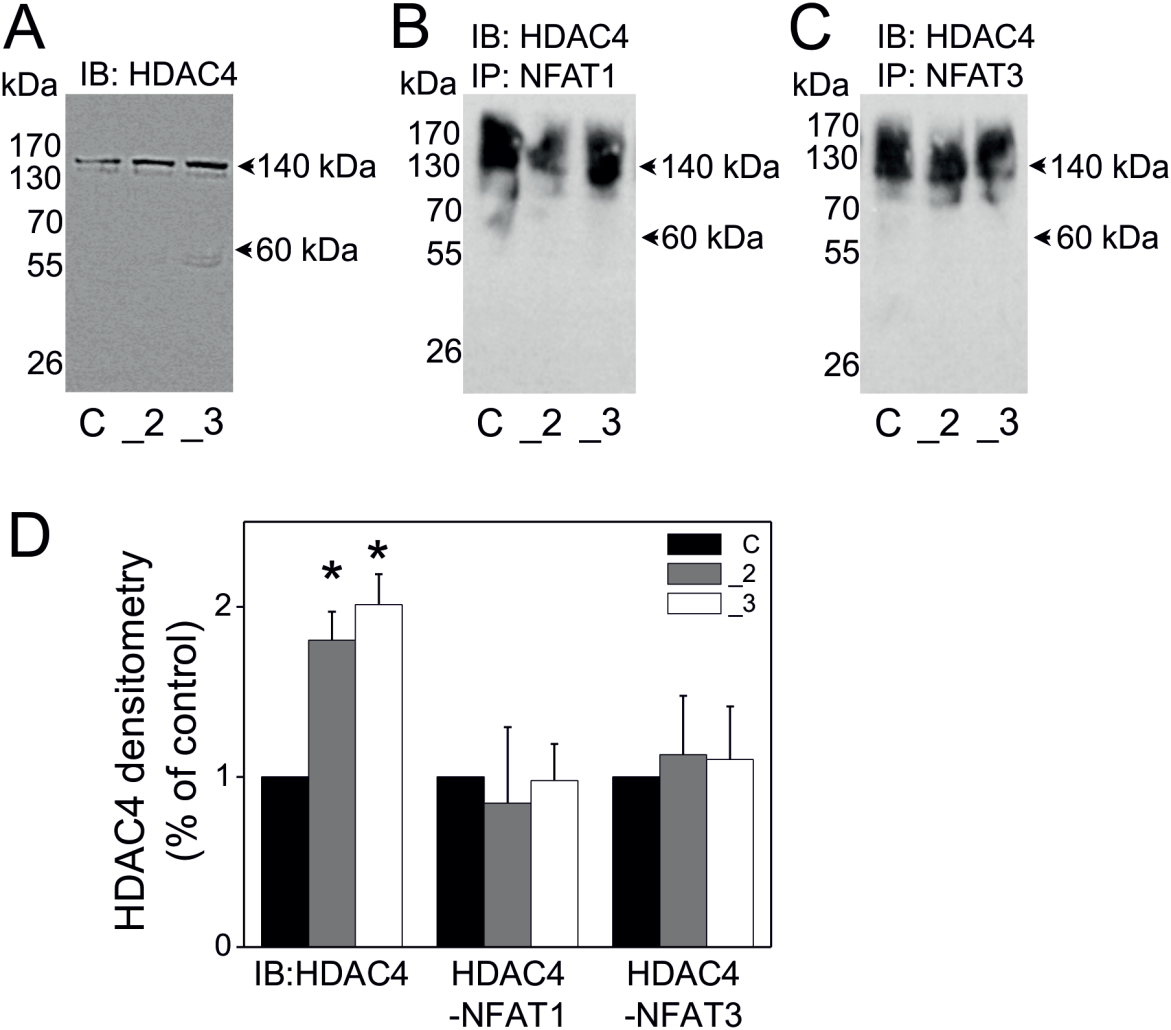
The interaction of NFAT1 and NFAT3 with HDAC4 isoform in PMCA2- or PMCA3-deficient PC12 cells. RIPA-total cellular extracts were subjected to immunoblotting to verify HDAC4 protein content and served as inputs of immunoprecipitation (**A**). The cellular extracts (inputs) were incubated with protein A/G agarose beads and with anti-NFAT1 antibody (**B**) or with anti-NFAT3 antibody (**C**) and the obtained immunoprecipitates were subjected to immunoblotting for HDAC4. All immunoblots and immunoprecipitates were measured densitometrically and expressed as % of control cells (**D**). Student’s t-test was used for comparison of control cells with PMCA2- or PMCA3- deficient cells. *P≤0.05, n = 3. Symbols: control cells (C), PMCA2-deficient cells (_2), PMCA3-deficient cells (_3).

As suggested above NFAT might work alone or in complexes with other proteins [64]. NFATs were found to cooperate with HDACs, where NFAT1c mediated HDAC-dependent transcriptional repression [57]. Moreover, both NFATs and HDACs were found to be involved in regulation of alternative splicing [41], [42], [49]. To check whether NFAT cooperates with HDACs in PC12 cells with different PMCA status we first analyzed the presence of various HDACs in total cellular lysates obtained from these cells. This analysis revealed that HDAC4 was predominantly expressed in all examined PC12 cell lines (Fig. 4A). Densitometry analysis showed that in the PMCA2- and PMCA3-reduced cell lines the amount of HDAC4 was significantly higher than in control cells (Fig. 4D). We have tested as well HDAC1, HDAC2, HDAC3, HDAC5 and HDAC6 isoforms, however due to weak signal and very low or residual protein level of these isoforms, and thus, due to low importance these data are not shown in this paper. To study the putative interaction between NFAT1 or NFAT3 and HDAC4 the co-immunoprecipitation assays were performed. These experiments suggested that NFAT might interact with the HDAC4 isoform, both in the case of NFAT1 (ubiquitous) (Fig. 4B) and NFAT3 (neurospecific) (Fig. 4C). The content of immuneprecipitates was similar in all cell lines, as verified densitometrically and expressed as percentage of control cells (Fig. 4D).

### Cooperation of NFAT with HDAC4 in Regulation of Alternative Splicing of PMCAs

**Figure 5:**
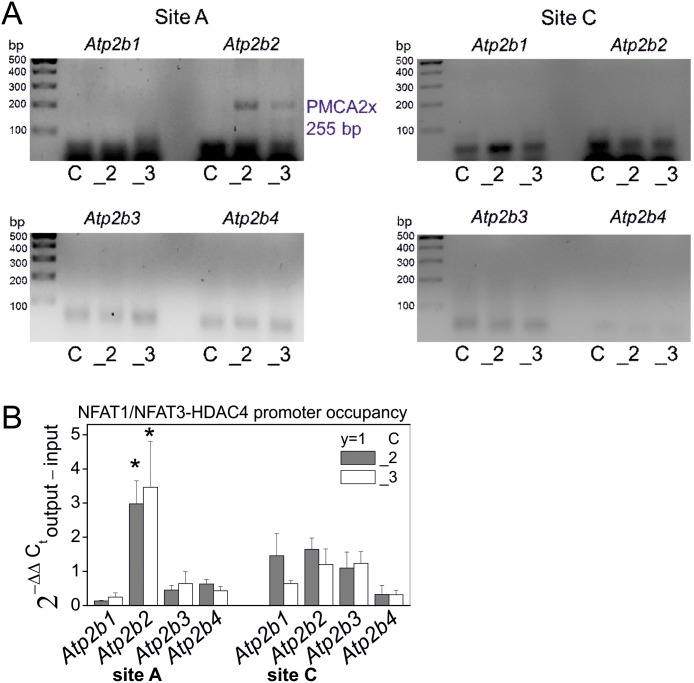
HDAC4-NFAT1/NFAT3 complex contribution to regulation of PMCA alternative splicing in PC12 cells. HDAC**/**NFAT involvement in regulation of alternative splicing of PMCA transcripts was analyzed by qPCR-chromatin immunoprecipitation. The analysis was performed for the splicing at site A and at site for four PMCA isoforms and the PCR products were migrated in 1% agarose gels (**A**). The qPCR data, were expressed as fold of change (2−ΔΔC) calculated from the difference: ΔCT of output (immunoprecipitated DNA with HDAC**/**NFATs) – ΔCT of input (total DNA) and statistics were calculated according to nonparametric paired Wilcoxon signed rank test at 95% confidence, where PMCA2-deficient cells (_2) or PMCA3-deficient cells (_3) were compared to control cells assigned to y = 1 value (**B**). Symbols: control cells (C), PMCA2-deficient cells (_2), PMCA3-deficient cells (_3), n = 3.

Regarding the protein interactions suggested above and formation of protein complexes consisting of NFAT1 and HDAC4 or NFAT3 and HDAC4, in the next step we examined whether these protein complexes may play a role in the regulation of alternative splicing of PMCAs. For that, binding of the NFAT1/NFAT3-HDAC4 complex to the splicing regulatory sites of PMCA isoforms has been studied with the use of chromatin immunoprecipitation technique with some modifications. Cross-linked chromatin and proteins were incubated with anti-NFAT1 and anti-NFAT3 antibodies. The DNA-protein complexes were further immunoprecipitated with anti-HDAC4 antibody. According to this protocol, generation of the PCR products of PMCA alternative splicing variants should be linked with the NFAT1/NFAT3-HDAC4 complex. Among all possible combinations of PMCA splicing variants only the PMCA2x splicing variant was detected under these conditions, suggesting that its generation might be related with HDAC4-NFAT1/NFAT3 binding to the splicing regulatory sites of the gene encoding PMCA2 (Fig. 5A), as shown in the supplementary material 2 (Fig. S2). The qPCR data, were expressed as fold of change (2−ΔΔC) calculated from the difference: ΔCT of output (immunoprecipitated DNA with HDAC**/**NFATs) – ΔCT of input (total DNA) and revealed that PMCA2x splicing variant generation was statistically significantly related to the NFAT1/NFAT3-HDAC4 complex activity, according to nonparametric paired Wilcoxon signed rank test at 95% confidence (Fig. 5B).

## Discussion

Our understanding of the regulation of the expression pattern of PMCA isoforms and their splicing variants remains incomplete. Only a few transcription factors involved in the regulation of the formation PMCA transcripts have been identified. It has been found that c-myc was able to repress PMCA4 expression during activation of B lymphocytes, which additionally resulted in decreased Ca^2+^ clearance, and interestingly, in an augmented NFAT level [65]. A similar research has been conducted in pancreatic β cells showing that c-myc expression has led to downregulation of PMCA1 and PMCA2 [66], [67]. Important data, showing that PMCA4 expression could be induced by NFAT1c in osteoclasts during the growth of bone mass, have been provided recently [43]. Furthermore, Kim et al have shown that PMCA-mediated increase in Ca^2+^ efflux prevented NFATc1 activation, forming a negative regulatory loop [43]. In our study we propose another novel role of NFATs. Namely, our results suggest that NFAT1 and NFAT3 activation may repress the expression of PMCA2, PMCA3 and PMCA4 isoforms, especially the PMCA4b variant. Moreover, we showed in our latest studies that overactivation of NFAT might be responsible for the repression of genes *Vamp1* and *Vamp2* in PC12 cells, thus indicating its role in regulation of the primary function of PC12, i.e. Ca^2+^-dependent secretory response [58]. Importantly, we showed that this coincided with a decrease in Ca^2+^ efflux, activating NFAT, and was accompanied by a dramatic arrest of the secretory machinery and a significant decrease in catecholaminesecretion. Summarizing, our data and the results of Kim et al suggest opposite NFAT functions and opposite interrelation between NFAT activity and PMCA expression. These contradictory scenarios are equally possible, especially during different physiological processes, such as growth of bone mass during osteoclast differentiation or catecholamine secretion by chromaffin tumor cells. Finally, it is worth to mention that NFAT has also been involved in regulation of the expression of other calcium transporters, such as voltage-dependent calcium channels (VDCC) [68], [69] or sodium-calcium exchanger (NCX) [70]. In general, downregulation or upregulation of PMCAs may be a common component of activating triggers in a wide variety of different cell types during various physiological process.

The biodiversity of PMCAs is based not only on the transcription of four independent genes but also relies on alternative splicing of the nascent pre-mRNA, transcribed from the DNA template wrapped on histones. Chromatin organization creates several points at which alternative splicing might be regulated, both at mRNA and chromatin level. Since our results suggested that NFAT was a repressor of PMCA transcription, we subsequently examined the possible role of NFAT in the regulation of alternative splicing of PMCAs in pheochromocytoma cells. We have undertaken these studies because of the recent evidence showing that alternative splicing occurs and is regulated co-transcriptionally [46]–[51] and because of the physiological importance of alternative splicing for the catecholamine secretion process. It remains undisputed that the functional variety of secretory proteins and calcium transporters depends largely on alternative splicing. For example, alternative splicing affects the formation of the exocytotic membrane fusion complex SNARE (SNAP (Soluble NSF Attachment Protein) Receptor) by altering the expression profile the following elements of this complex: synaptosomal-associated protein 25 (SNAP25), syntaxin 1 and synaptobrevin 1 [18]–[21]. Furthermore, there are several examples of alternative splicing of mRNA transcripts of several calcium transportersincluding PMCAs, responsible for calcium removal, as well as channels and ions exchangers responsible for calcium influx, i.e. VDCC [22], [23], [71], [72] and NCX [24], [25], [73]. The above examples underline the importance of the alternative splicing process in catecholamine secretion. Since in this report we propose NFAT as a transcriptional factor involved in splicing regulation, it is essential to discuss the relationship between transcription and alternative splicing. For instance, it has been shown that the process of alternative splicing depends on the gene promoter driving the transcription [74]–[77]. Moreover, alternative splicing might rely on the recruitment of transcription factors or co-activators to gene promoters [38], [39]. It is very likely that NFAT binding to the promoter regions of NFAT-regulated genes may alter alternative splicing mechanisms by trans-activation or trans-repression. Some RNA sequences that are called splicing recognition sites and are located in exons or introns, might stimulate or block splicing by binding specific splicing regulatory proteins such as serine/arginine rich proteins, snRNP or transcriptional factors [36], [78]–[80]. Based on our bioinformatic analysis and the relationship between inhibition of NFAT transcriptional activity and PMCA expression pattern, it is very likely that NFAT might bind to some splicing regulatory sequences. Moreover, NFAT has been found to bind not only DNA motifs [81], [82], but also to interact with some RNA sequences [83], [84], usually acting as NFAT repressors, like the non-coding RNA repressor of NFAT (NRON) [85]–[87]. It remains unclear which of the putative sequences in the PMCA coding genes that were showed in our *in sillico* analysis inhibit and which stimulate NFAT activity. Our study demonstrated the relationship between NFAT and PMCA expression pattern, based on experiments with inhibitory peptide 11R-VIVIT at physiological level. Distortion of this relationship results in disturbed calcium homeostasis and disturbed catecholamine secretion in pheochromocytoma. More studies with the use of the selected sequences are needed in the future to establish the exact regulatory sites for NFAT in PMCAs genes, regarding both regulation of expression and alternative splicing pattern.

Furthermore, in this report we found that NFAT might cooperate with histone deacetylases; more precisely we suggest an interaction of NFAT1 and NFAT3 with HDAC4. Our results are supported by many other data. For instance, it has been found that NFAT1 may cooperate with HDAC4, HDAC5 and HDAC7 in chromatin remodeling [57], [88], [89]. More precisely, it has been shown that NFAT1 might act with HDAC4 in a repressive complex during osteoblast differentiation [90]. Interestingly, both NFATs and HDACs have been found to be involved in the regulation of alternative splicing. NFAT1 has been suggested to participate in the regulation of alternative splicing of the allograft inflammatory factor-1 gene [41], and of synaptotagmin-like 2 gene during activation of murine T-cells [42]. HDACs have been proposed to contribute to the process of alternative splicing by regulating co-transcriptional spliceosome assembly [51]. Regarding the data provided in the literature on the functional interaction between NFATs and HDACs and given that both NFATs and HDACs are involved in the alternative splicing process, the next question addressed in this study concerned the role of this cooperation during alternative splicing of PMCAs. Our data suggest the contribution of the presumed complexes, NFAT1-HDAC4 and NFAT3-HDAC4 to alternative splicing of the mRNA transcript of PMCA2. More precisely, these complexes are supposed to enhance the generation of the 2x variant. Based on our bioinformatic analysis we suggest that the mentioned protein complexes might occupy some parts of the promoter region of the PMCA2 gene, and modulate the availability of some exons to alternative splicing. This is in accordance with the influence of some transcription factors on the formation of the nearby spliceosome. On the other hand, this might also suggest binding of the NFAT-HDAC complexes to the intronic splicing regulatory sites, preceding the excluded exon 7 and exon 8 but not to the intronic fragments of the gene preceding the included exon 9, resulting in the removal of exons 7 and 8 of the PMCA2 transcript leading to the formation of PMCA2x variant. The involvement of NFAT in this kind of splicing regulation is highly probable regarding our bioinformatic analysis showing numerous NFAT-specific motifs before exons 7 and 8, but not before exon 9 of the PMCA2 transcript. Generation of PMCA2x transcript with the contribution of NFAT1/NFAT3-HDAC4 complex could be a compensatory mechanism in response to experimental downregulation of PMCA2 or PMCA3 isoform.

It is worth to underline that the splicing recognition sites in the intronic/exonic regions of PMCAs genes were enriched in NFAT binding motifs (5′-GGAAA-3′ or 5′-TTTCCC-3′). PMCA2 and PMCA3 coding sequences contain relatively the highest number of NFAT-specific motifs, while those of PMCA1 contain a lower number of these motifs. Interestingly, NFAT binding elements were found in the proximity of the TATA box motifs (5′-TATAAA-3′), especially in the case of PMCA2 splicing regulatory regions before the spliced exons 7 and 8. This is similar to the interleukin-2 (IL-2) gene promoter [91] or COX-3 gene promoter [92]. Another example of NFAT connection with TATA box-proximal region has been given for the regulation of the promoter of the gene coding for p21 [93]. This data further reinforce the possible involvement of NFAT in regulation of the expression of PMCAs, especially PMCA2x.

Finally, an issue worth to be stressed is that an increased content of the endocrine-specific PMCA2x splicing form has been particularly observed upon deficiency of neurospecific variant PMCA3x. PMCA2x and PMCA3x variants exhibit the highest efficiency in calcium removal and the highest affinity for calcium ions among PMCA isoforms [64], thus it is obvious that a decrease in the content of one of these forms might be compensated by an up-regulation of the other. Such modification in PMCA composition probably serves to retrieve the affected calcium signaling and to rescue a disturbed secretory response in PC12 cells. Several examples of PMCA compensatory expression has been provided, including the compensatory expression of PMCA4 upon reduction of PMCA2 [94]. These data further support our results, because we have similarly observed an up-regulation of PMCA4b in cells with a reduced PMCA2 content, as well as in cells with a reduction in PMCA3 content. Interestingly, the expression of the PMCA4a variant was increased upon NFAT inhibition. This suggested a putative involvement of NFAT in maintaining the balance between expression of PMCA4b (upon NFAT activation) and PMCA4a (upon NFAT inhibition). This is in accordance with the results showing that NFAT might occupy the promoters and might be responsible for the control of the expression of PMCA1 and PMCA4 isoforms in osteoclasts [43]. Summarizing, all changes in PMCA composition were accompanied by activation of endogenous NFAT1 and NFAT3 suggesting their involvement in the control of PMCA expression pattern and, via interaction with HDAC4, in the control of alternative splicing of the PMCA2x variant. Thus, NFATs-HDACs complexes may play a role in the fine tuning of the process of catecholamine secretion via determination of the optimal expression profile of calcium transporters such as PMCAs.

## Conclusions

The aim of this study was to verify whether the alternative splicing pattern of PMCAs in PC12 cells might be controlled by a protein complex composed of NFATs and HDACs. In this report we suggest that HDAC4 possibly operates in a complex with NFAT1 or with NFAT3 that might be involved in regulation of alternative splicing of PMCAs. This complex probably contributes to the generation of the splicing variant PMCA2x. Moreover, this was probably a compensatory response to altered composition of PMCAs, namely to experimental reduction of PMCA2 or PMCA3 isoform. Furthermore, this was linked with an altered calcium homeostasis. We propose that NFAT1 or NFAT3, in complexes with HDAC4, might occupy regions in the putative splicing regulatory sites in pre-mRNA of genes coding for selected PMCA isoforms.

## Supporting Information

Figure S1 **Protein level of PMCA1, PMCA2, PMCA3, PMCA4 in control, PMCA2- or PMCA3-deficient PC12 cells.** Protein level was analyzed by immunoblotting as described in Materials and methos. 25 ug of cell lysates was used for the presented immunoblots for PMCA1, PMCA2, PMCA3, and 10 ug for PMCA4. Protein bands were quantified by densitometric analysis, i.e. the content of PMCAs was standardized to Δ-actin level in all cell lines and then normalized to control cells, expressed as y = 1. Student’s t-test was used for comparison of control cells with PMCA2- or PMCA3-reduced cells. *P≤0.05, **P≤0.01; n = 6. Symbols: control cells (C), PMCA2-deficient cells (_2), PMCA3-deficient cells (_3) **(A)**. The antibodies used are presented in the table and the predicted PMCA forms, recognized by the antibodies, are marked as ‘expected gel band size’ **(B)**.
(TIF)

Figure S2 **Detection of NFAT-target sequences in spliced regions of exon/intron junctions of genes encoding PMCA1, PMCA2, PMCA3, PMCA4.** Selected gene regions or selected single nucleotides in exon/intron frames of genes encoding PMCA1, PMCA2, PMCA3, PMCA4 were assigned as sites of recognition during alternative splicing according to ensemble database (http://www.ensembl.org). These regions were next analyzed in terms of the presence of NFAT-target sequences (TTTCCC and GGGAAA), as well as TATA box motifs (TATAAA).
(PDF)

Table S1 **Antibodies used in this study.**
(DOCX)
