# Supplementary material for: NFAT1 and NFAT3 Cooperate with HDAC4 during Regulation of Alternative Splicing of PMCA Isoforms in PC12 Cells
Source: PLoS One. 2014 Jun 6;9(6):e99118. doi: 10.1371/journal.pone.0099118 (PMC4048221; doi:10.1371/journal.pone.0099118)
Supplement: Figure S2 — Detection of NFAT-target sequences in spliced regions of exon/intron junctions of genes encoding PMCA1, PMCA2, PMCA3, PMCA4. Selected gene regions or selected single nucleotides in exon/intron frames of genes encoding PMCA1, PMCA2, PMCA3, PMCA4 were assigned as sites of recognition during alternative splicing according to ensemble database (http://www.ensembl.org). These regions were next analyzed in terms of the presence of NFAT-target sequences (TTTCCC and GGGAAA), as well as TATA box motifs (TATAAA). (PDF) [file pone.0099118.s002.pdf]

Splice region in PMCA1 gene (ID: ENSG00000070961)

Legend:
GGCGTGTGGGTGTGG – exon
TTTCCC – NFAT binding motif
GGGAAA – NFAT binding motif
TATAAA – TATA box
alternative splicing region
R **– altered nucleotides recognized during splicing**

SEQUENCE BEFORE SPLICED EXON 39 bp

Intron 1 – 2

```
GAGGGGCCCCAGGTGAAGGGCGCCCGCACTTCTCCTGCGGCAATCATCGCTCTCCTGCCT
CCACACGTCGCGGCAGCGGCCCGGCCCCGCGCCGAAACAGCAGCTGTGACTGACGGAGTC
ACCTCCCCGCGCGCCTGGCCACCCCCTCCTCCCCCGCCGCTCCCTCCTGCACCAACACGT
CATGGAAACACGGACGTCAGAGCCTCTCAGCCAATCGCAGGCTGCGGCAGGAGGGAAGGG
GCGCGCTGGAGGGGGCGGGGGCAGCGGCAAGCCAAGGCGCTCAGGGGAAGGAAGAAAGGG
AGGGGCGTGCGGAGGCGCAAGTCCCCACCTCCGCCCCTCACTCCGGCCACCCTTTCCCGA
CCGCGCGGCAAGTTCTAATCGCCAGGCGCGCTCGCCAGCCGAGGGCGCGCTCGCCGGG
TCCTCCCCTTAAACGCCCTCCCAGTGCCGCTCAGCTCCCAGTCCCGGTCCGAGCCGCGGT
TCCTCCGCGCGCGCGCGCGCACTCGCCCGCCCCCAGGCTCCCCGCGGATCGCGTCACG
TGACCGCGCTCAGCCAAGGCGGCAGCGGCGGCGGCGCGAGCTTCGGCGGCGGTGGCGGCT
GTGGCAGCGGGAGCAGCTGCGCCGCCAGCGTGGAGCGGGAGGCGTGTGGGTGTGGAGAAA
AGGGAGCGCCAGACCGGAACGGGTGAGCGTTCTCCTCCCCCTGCCTACCCCGGCAATCTT
GCAGGCCACCCGGTGCAGACGGGGTCCCTGCCCGGAGCTGCGCGCAGCCCCCTCGTGCTC
GGCCGTCCGCTCTCCGGTGCCCGCGGGCCCCGCCCCGAGTGGGGCGGGCTGCGGCGCTCA
CGGAGGTGGCTGCACCCCGCCCGCGGCTGCTGTTGCGGACGGCGGCGTGAGGCCGGCTGA
GCTGCCCGTGCCGAGCGAGCGCGGCGCGCCCTGTGTGCCGCTCTGCGGAGCCGAGGAGGC
GGGTCCTGCGGGGACGCGGGCGGCCACTCGGTGGCTGCCGGGCTCTGGGCGGGGACGGCC
```

Intron 5 – 6

```
CATTGTGTAACAACTGTTATAGAAATGTGAACTACTTCTGCATAATTTAGCATGGCTGTA
TTCATTTCTTTTGGAAAGCTTTAATTCCCATTTTGTTCCTRCAGATAAGAAACAAGATGG
AGCTATTGAGAATCGCAACAAAGGTAAATTTAGGAGGACAAAAGTACAGTGAGCTTGTTT
TCATTATATCCAAAAATCTACTGTATACCATATTTATTCAAAAGTAAGATACATGTGGCA
GGGAAAATCAACAATATACGTTAGCTTGGGTTAACCTTTCTTCCTGCCGGTGTCTTAGTC
```

SEQUENCE BEFORE SPLICED EXONS 154 bp

Intron 19 – 20

```
GACTTTTAACTGATTATGAAAGAATGTGTCTATATGCCCCTAAGCTCCTTTTTTCCTAGC
TCCCCCATCCCCCACTTCATCTCAAAATGCTATTCTTGCCTATATGCTGTGTAAAAAGTG
CCTTTTTCTTTTTATATGTTGATAGATGGAATGGTCCTTTCTCTTGTTCTCTCTCTCTCT
TGCTGAGCAAGCTGTCACAATCTCTGATTCCTTGCAGATGGATGTAGTGAATGCTTTCCA
GAGTGGAAGTTCCATTCAGGGGGCTCTAAGGCGGCAACCCTCCATCGCCAGCCAGCATCA
TGATGTAACAAATATTTCTACCCCTACACATGTAGTGTTTTCCTCTTCTACTGCTTCTAC
TACTGTGGGGTGTGAGTGTGTGTTCCTAAGTGCATGAAATTAACATTTCCTACTTCACAC
ACCTAACGTTTCTCATTTTCTCCTTAATGTTTGAATTCATTGTTCAGGCTTTTTCATAAA
GCTTTCTTTTCGTAAAGTGGATTGAGACCTCAAAGTGTTGTCGTTGTTGTTTTATTTCTT
```

Intron 20 – 21

GCTGAACTGGCCTAGTAACGTGCTGAAACTAACCAGACATATGGCTTAAATAGATGTGTC
AGACAAAGGAAGCATTAACCTTAATCTTATGGAATGTTTTATTCAAGTGAAGTTAGGGAT
AAAGTAAATGATCGTATGTTTTTGTCTTCTCTTGACTTCCAAGGAACCTGGAATTGGGAG
CCCAGAAACCTAGGTTTTAAGACTCTTTAGCACTAATTAGGAGTTCCTTAACAAGTCAGC
CACCCATCCTCTCTGTTGCTCAGTTTTATTATTTATGAAGTGGTTAGAAAAAAAAAATCT
CTAATATTGTTCAACAAATTCAGATA**GGAAA**AATCTTCTCAGAATTGAAGTTTTGCTAAG
AAGGGATTTAATCTTTCTTTGCCTGCCACCGGTGTATGTGTGTGATTGTGTGTGCACATA
TACTTGTTGCCCTCACCTTTACACTCTGCCCCTCTTAGAGCTTCACTAACTCTCAAATCT
CTAACTTAAGTTTACACTTTTCTTCTGAGCTCTAGATATTTTCCAAATTTAACATGTTTA
AAAAGTACATTTATTTCCCCCCGATTCTTCACTGCTCACTTTTATACCGACTGATTACAA
AGAAAAACAGAT**GGAAA**ACACACACATACACAGAAAGCTTAACTGTTGTTCTATCTTCAG
TAGCAGCAGCACCAGCAACCTTATCAAATAAGCAACATCTTCAGGGTTGTCTTGAACAGT
TCTCATCACCCATCAAGTTTGCTGGCTGATCCTCTAATTTGTATTCTCCTCCGTGCCTAC
GGGCTGCTGATACCAACAAATTTTTTTTTAGAAAGTTATCTTTTTGCATTTTAAGCTTTA
CAGTTTGTTATTAGGACAGTCAGGATACTTGCTGAATGTTCACATGTATAAAATGTGAAT
TTTAAGACTTCACAATAAAACACTGAATGAATTACTATAACGTTATATTTAGCAAACATT
GTTTACATTCCATATGTAGAACACTTTACATCTTAGTAAAGGGGAAGAGGAACAAGAGCA
ATAAAAGAAGTTGAAAAATATTTGCTGCTATTCATCAGTCAAATTAAGGAACTGAAGTAC
AAGCAATCAAATAGAATGGAATTATAAAAGATGGTAAAGCTGCAAGACATTAATAGAATA
GATGCTTGCTGCTCCAAGTGTGGATCATGGACCAGCAGCTTTAGCATCACCTTGGAGGTT
GTTAGAAATGCAGGACTTTAGGCCCCTGCTGAATCAGAATCTGCATTTTAACAAGATCCC
CAACTAATTTGTATGCACATTAAAGTTCGAGAAGCCCTGGCCTCGATACTTGTGACCTAA
CATTGTTTTAAGTTTGTAGTGTGCTAACGAATTTAATCTCCCACAACAACCCCGTAAGGC
AAGTACCATTGCTATTTCCCATTTTATAGTTGAAGAAACCTGTTTGACCGAGGTTCCTCA

PMCA1: 6 NFAT motifs

Splice region in PMCA2 gene (ID: ENSG00000157087)

Legend:
GGCGTGTGGGTGTGG – exon
TTTCCC – NFAT binding motif
GGGAAA – NFAT binding motif
TATAAA – TATA box
alternative splicing region
R **– Altered nucleotides recognized during splicing**

SEQUENCE BEFORE SPLICED EXONS: 33 bp, 60 bp, and 42 bp

Intron 1 – 2

GTGTGTGTGTGTGTGTGTGTGTGTGTGTGTGTGTGTGTGTGTGTGTCTCACTACCAGTCT
ATCAAGGTAACTGAAATATCACCATTAATTCTTTTTCTCATAGAGAACTTTCTGCTAGCT
CTCTAAAGGAATGAAAATAACCTTGGGTAGGTAATGGACAGGGAGAATCCTAAGTCATC
AGCCAAATTATTTATTCATTGACATCTCCATTCATCCATTTATTCACTCATCCTTTTCTC
CATCTATTCATTTATCAGCCTCTCTAACTACCAATTCATCCACCAGTCCATCCATCCATC
CACCCATCCATCCATCCATCCATCCATCCATCCATCCATCCATCCATCCCCTTCTCTCTC
CATCTGTATCTCCATATATCCACTTTTCTTTCTAGCCATCCATTCATCCATCTATCCTTT
CACCAGGTTCTCACTAAGCATCAATTCTGTGACTGGCTCTGTGGGGTAGAGATGAAAAGG
CTCACTCAGTTCCAGCCCCTGCAGATCCCCCAGGACCCTGGCAGCAGCAGATCTATGAGC
CCACA**TTTCCC**CCTGCTTGTGAGGATCACACCAAGAGAGACATTCACATCCTGGCCTCAC
AAAGAAGAGATGGCAAAGT**GGAAA**AAGGCCGGAGTCCCCAACCCTGAGCCCTTTACCCTG
TCTCAGTCTTTTATAACGAGGTGTGTGTGCTTGGGCAATGCCTCTCTCTTCTGGGACTCA
GTGTACCCATCTCTGCTGTGGGGAGGTTGGACTAGATGAGAG**TTTCCC**AAACTCCAGTTG
TTTC**TTTCCC**TCCCTCCACACCTGGAGATCAGAACACCTGTACTAAAAAGTACTGAAAAT
CTTTATTCAAATGAGCTCACTTTTTTTCAACCAAAATAAACTTATTTTAGAGTGAAACTT
TATTGCTACCATAAAT**GGGAAA**AAAATGGTAACAGCTGTCATAAATAGAAAGTAACAATT

Intron 2 – 3

AAGCTGACCTCACAGCTAAGCAGAGTGGGTGGTCGCCTTAGTGATGTGAGTCGAGGGGTG
CCAGGCAGCTCCCTCTGCTGCAAATCCCTCCCACTCACCAAACTGCTGCCGCCTGCAATT
CCACAATGAGCACTTTAGTCATCAAATCATAAAATGTCAGCTTGTAATTAGCCTTGGAAC
CTGTCTAGTTCAA**TTTCCC**AGCTCAGAGTGCTCTTGGAAGATACCTGAACGGGCCCTTTA
TTTTAGAGTGAATTAGTGGCAGAGCTGGAACTCGAGTCTGACCCTGCACAGTGAAGGACG
CTAGAGGCATCTGCAGCTTCTTCAATGGGAAGGTGGGTTCTGAGTCACAAGATGGGCAGC
TCCCCAAACAGAGGAAGGGGTTCAGACATCAGGCATCCAAAGTAAATGACCAGTGTCCTA
GTATTTATTTAGCCCTCACATCAAAATGTCAAC**TATAAA**GAAAAGAGGCGACAATATTCC
GTTGAACGTTGCCTTCTCTTCAATCTAAACTAATTCATCCCAAGTAGGATCCATTATCTG
ACTGTCTCATTATAGTCACACT**GGAAA**AGCACATCTTGTTGT**TATAAA**TGACTTTCATTT
TATTTCTCTTCTTTATTACAGTTGGGGCATCATATGCCTTTTTGAAATTATGTGTATTGG
TAGGTTATATGATGTGTGAATTTTCTTGGAAGATAAAGAAAAAAAGTAAAATGATGTTGG
ATCCAATGGGACCAAGAGCCACCAGCACAGTGGATAGAAACAAAGGCCCTGTACTGAATC
CTGCCCTGCCTCCTAATTAGCTAGGACCTTGCATATGAGCATGAACATCAGCTCTTACCC
CGGGTCTCTGAAGTCATTTATTGAACTTGG**TTTCCC**CACTAACTAGTGGGTGAACAGTGG

Intron 3 – 4

CTACCGCGCACCAACACTGAGCTGGGCAGTGGGATTTTACAGCAAATGAAAGCAGTCTAG
GGGGACTAACGTGTCCAGGTTTGTCTGGGACATTCCTGATTTTAACACTGAAAGTCCCAC
ATCCCAGGACACCCCTCAGTCCGAGGCAAACCAGGACAGTTGGTCATCCTAACACAATCT

CTGCCCTCATGGATTTACAGTTTAGTGGAGGGACATAGACAATAAGTAAATATATAACAT

Intron 7 – 8

GACTGCAGAAGGCCCAGTCTGCTACTCGGCCCGCACTCTCTCCATTACACTACCCTGCCT
CTTCTCCATGAGAGGCAGCGGGGTGTAGTGGATAGAGCACGGGTTCAAGTCCCGGCTCCA
CCACTCACAGCTGTGTGACCCTGGGCCATCACTCAAGCTCTCTGAGCCAGAAAGTGGTGG

Intron 8 – 9

AGAAACCTCCCTCCTGAGTCCCCTGGAGAATCAAGAGTTGCCCGGCTGGCAGGCCCCACA
GCTAAGGTGCGGCCAGGCCTGCTCCTGCTTCAGGGCCCTCCCCAACCCGTATCCCCACAC
AGAGGGATGCAGTCAGCACGGCCCGCCCTTCATCTGTGACATTAATGTCTGTGGCCTGTT
GCGTCTTCGTGATGACCGACCATGTGTTGTCTGTTCTCTGTTGTCTGTTAAAATTCTTTG
TCGTGTAR**GTAAAATGCAGGATGGCAATGTGGACGCCAGCCAGAGCAAAG**GTAACCMTTT
TGTTTTAACCGGCGTTTGTCGTCCCTGAGGCCCCTCCCCACCGGCTCTGACAGTCACACC
AGAGACAGAGACTGGCAAGGGCCAGAGGCAGACACATCCACTCCTACAGGTGTCTGACGA
CCTTGGGGGTGTAGCTGTGATTTGCATGGTGCCAGGCCAAATAAAGAGGTGTCCACCGGT
GAGGAGGGGGATACCCATGGGCCAGCATCTCCCTAGCCTTTGAACTCTGCGCCCCATTCA
GCTGAAGACTTTGAGATCCTTAGAGGTTGAGGGGTAGGGAGTTTGGCTCAGTCTCTGTCC
AAAAGGGTCACGGGCCCTCCAGTCCATCCTGCATGAAGAGGCCACGCCAACTGGGATAGT

Introns and enxons 7, 8, 9

ACCTGCACTCCTGTGAGATGGAGATGTCAGAAAAATAGCCTCATGTAGAAGGAAGATGGG
GAATGTGGAAGGGATGTGGCAGAGGAAGCCTGGGGGAGCAGTCCAGCATCTCAGGCCACC
ATGAGCAGCATGGCCTCAGCTGGGCCCCCTCTGCCCTCCCATCCCATCACCTCCACTAGG
AACATTGCTTAGAGCCCGCCACATGTCCCCATTTCAGGATGACCCTAGGTGTATGGCCAT
GTGTGTATGCACATGTGTGTTTGTGTGTGTGCGCGTGCGCATGGGCAGATGAGGCTGTGT
GCTTCTCCTTCATTAACATGTCTCAAAAATCATCACAYAG**GTGTGAAGAAGGGGGATGGC**
CTTCAGCTACCAGGTACAGTAAGACACCCTCAGGGTAGATGGGCTTTTAGAAATAGCAGC
AGGCTTTGGTCATTGGGCCCTGCCCCTAGACCACCAGCACACAGCTGGAGGAGGCCACAT
GCCTGTGGGTGGCTCCACCCCCCAGACCCTCCTTTGTGCCCTCCTGTGATGCCAGCCCTG
TGGGACAAGCCAACACCCCACTCCACCCTCTTGCCCTTACAGTGCCTCACCCTACCAGCC
AATGCCAAGTGGTACCACCCCCATCTCTACACCACCCTTAGCCCTGCGTACCAGCATGCA
AAGCTTGCTCACCATCATAGGACCCTCCCCTCAGGCCCTGCTCTCCCCACCAGCACCCCT
GGGGAGCCCATGCCCTCACGCAACAGTGTGCTTCACCCAGTGGGGCCTCTTCCTGCCCAT
AGAACACTATTCCCTACCCACAAGAAAGAGCAAAAGGTGTGAATTTAGCCATCTCATATC
ACTTTTAATTTCTAAATTAAGAAAAATTCACTAGTGCCTTTTCCATCCCATATACAAAAT
GCAACATGCTTGATGTCACCTTTTAAATTTGGGGGGCATCTCACATGGGAGTTCCCAAAG
CAGTCTCTGGAATGCCAGTTAAGGAAGGAGGCAATGGCCTGAATCAAGGGCATGAATCCA
GTTAAGTCAAAGTCATCACCATTAGCAAATGTTCTTCCAGAACACCCTGATGCACAGAGA
TGGTGGTCATGGAGAATACTCATTCTGAGTGGTCCGTGCCCACGCTGCACTGCCTAGTGA
ATATTTTTCCACTCCTGGCCCCCAATGGGCAGCCAGATCTCCTCATTTCATGGAGCCCTC
TCTTCCAGGGGACTCTCTTGACTGGTTGGACCAGCAGGGTCCAGACCTCAGCACAGACCA
GCACCATCCCAGCTAACCACCCTGGTCATCCACCATGCTTTATTTGAAAGACCCTCCATG
TCTGAGTTTCTCCCCTCAACCTCTGCGGCATCTCTATGGAACATTTCACCCCCACCCCAA
ATTTGAGTTTCTTGGTTTCCAAAGGATAATTTTTTTTTTTAAGAATAGAGTCATGTTATC
TCCCCACAAGTTGGTTCATGTCTTTGTCGTTGTTGCTTTTCTCCATTGTGACACTGCATT
TTCCATCCCTCCCACCCYSSTGYAG**CAGCAGACGGTGCGGCAGCTTCAAATGCTGCAGAT**
AGTGCGAATGCCAGCCTAGTCAATGRTATGTCTCCCACGGCGAGCTCCTGGTCATGGTTG
GATGGGCGTCTCTCACCCCCACCCCAACTTTGGGCTTTCTTTCCTTGTTCTGGACTGCAC
AGATCTCGGATGTGCATGGGCCGCTGGGACAGTCACCTTCTCACATGGGGCAGGCGCTTA

AGACAGGAGCCACAGCCGGGGGAGTCAGCTCTGCAACCTGTCAGCCTCACAGCTGCCAAA
AAGGTGCTGGCTCCTGTCGAGCCCTTGACTCCCCATGGAGCCCAGATAAGAGACGAGACT
TTGGCTTCAGATTGGCTCCCAAGAAGGAGCGTCCCACCTGGCATGGCCCTCTCCTAAGTG
TATTCCTGGCCCTGGCTTTGGGAAGAAGGCCTGGGATCAGGGCTTGGGCTGAAGCTGGCA
TCTGGGTCCCCAGCAAAGGCTCTAGTCCTCGACTCTAAAATTTCCTCGAGGCAAGGGGAC
CCAGGCCGGGATTCAGCTCCTTGGCCCTGTCCTGTAGCATGGCTGAGCCAGCAGTAAACA
GTTTTTCCTCTTCTCTTTCACGGAGCCAACGGGCAGGGGGTTCGCTTGGGCTCGGGCCTG
GTTCTGTCCTTCGGATGTGGACTGGTGTTTCTTGCTGTTGCCTGTGCTGGAGCGTTTGTC
CTGGGTCACAGGGGTGGAGGGTGGGGGAGAGGGGCTGGCACTGTGTACTGGTGTCTGCAT
CTTAATCTCCTTACACGCAAGCAAGAAGGAGTTCCATTCACTTGCCCCCAAAGCCTTTAG
AAGATTTCGGATTTCTATCCCCTTCTGGCTTCAGAGCAACAGACAAGGCTTTTGTTTACT
CAGATACCACCCCTATCCCCAAATAAAGTGACATAATAGTCCCCCAGGCTACTCCTGAAC
TGTTGAGAGCCATTAGGGCTCCATCATGAAAACATTGCCCCAGAGGAGCTGAGGGCAGGG
GAAAATCTTTTTCCCACCGTCTCTGCATACCAGGCACTGGGCTGTGGGTTTCTCATCCAT
TATATCATATCATTCACTTCTCACCACTCCTGCAAAATATGGAGACTTGTTCCCCGCAGC
CTCCCATCCCCTGCCCTAGACCTCTCCCCAAGCCACCTGCATTCAAAGTGCCCAAAGATG
GAAGCACCTAGTAGGTGTCGGGGCTGAGTAAGGCACTTCTGCGAGCAGTGCCTTGCCAAG
AAGGACTAATTCTGGTATGGCTCTATTTCAGGCACAGGAGGAGGAAGGATGACAGCTTGG
GGAGTGGGAGGTGTCATGCCTCAGGGAGGGGTGGCTGAGGGTGACAAGGAGAACCCATGT
GGGCAAACCCATGGCTGAGAGAGAAGGAACCTAGAGGGGGCACCCACTTCACCCCGGCAG
TGAGCCTGCTGAGCTTTGTGAACATACAGACCCCATCCAGGCTGGCCAGTCTCAGAAAAA
AGGGCAGCTTTACTTCCAGACTGCAGCAGAAAACCCCAAATATCCCAAGAGAAACTGTAG
GAAACGTTCATTTCTTGACCACCTACTGTATACCAGGCACTGCTGAAAGCATTTCATATT
CACCATCTCATTTAATCCTCATGACACCCCCATGATACAGGTTTTATTGTCCCCACTTTA
AAGATGAGGAGACTGAGGCTCAAGGAGACCAAGACACTAGCCTAAGGTCACAGAGCTAGA
AAGTAGCAGAGCTGGGATTTGAACCCAGAACCCAGACCTGGCTGGCTCTAAAGTGTGTGC
TTCTCCTCCTGATCCAGGATTTCCCAGTGGAGTGTGTCTTGTAAGTTTCACAAGGATGTT
AAGAGGGCTTTACCCCAAGAAAAGGATTCTGTGGTCAAAGATGTTTGAGAACCACTGGAT
TGAATTGATCTCTTCCTGGTTACAGAATTAGTCCTGGTTACAGGACTGCTCAGAGCCTTG
AATATGCTGGTGTGTAGCGGAGAGTTACAGAGTCACATAGAGTTTCCTAGGCTGATTTGA
CTGCAAGACCTTTCCTGGCTGAATCCTGTGGAATTGGTCTGCCCCATAATATACTCTGGG
AAATAATGCTCTAAGCCATTGCTATTCAAAATGTGATCACAGGACCCAAAGCCTGCACAT
CACCTGGGAGCTCCTTAGAAATGCAGAATCTCAGTTTTTTATTTTATTATTATTATACTT
TAAGTTTTAGGGTGCACACGTACCCTAAAACTTAAAGTATAATAATAATAAAATAAAAAT
TAAAATTAAAAAATTAAAAAAAAAGAAATGCAGAATCTCAGACCCCACCCCAGACCAACT
GAAGGAGAATCTGCATTTTAACAGGCTTTCCAGACTCCAGGTGAGAGCACATTACAGGTG
GAGAGGCACAAGGTGGAACCTTGCCGATTGAGAGAGAATTAAGTGTTCTTTCCCCTCCTT
CCTCTGAGCCAGATCACAATGACTTGGATAT**GGAAA**AAAAAAATTAGGGTGATAATTTTT
CCCTTTGACCCTGCTCCCCCAAGCCCCCAACTGAAAAATGGGTGTGGTCCATTCATCTTG
GAAGCATTATGATTGCCATTATTATTTAGTTATTTTTCTTTTACATTACTAGTGTGTCAC
TATGTAATACATGACCTGTAATCATGGTTATAATCACAGTTAATTATACATCATGAGGCC
GGGCGCAATGACTCATGCCTGAAATCCCAGCACTTTGGGAGGCCAAGGCAGGTGGATTGC
TTGAGGTCAGGAGTTGGAGACCAGCCTGGGCAACATGGTGAAACTCTGTCTCTACTAAAA
ATACAAAAAAAAAAAAAAAATTAGCCGGTCATGGTAGTGGGCACCTGTAATCCCAGCTACT
TAGGAGGATGAGGCAGGAGAATTGCTTGAACCCAGGAAGTGGAGGTTGCAGTGAGCTGAG
ATCATGCCACTGCACTCCAGCCTAGGAGACAGAGTGAGACTCTGTCTCAAAAAAGAAAAA
AAAAATTATACATCATGGGCTGGGTACAGTGCTTCATGCCTATAATCCCAACACTTTGGG
AGGCCGAAGCAGGAGAATCACTTGAGGTCAAGAGTTTGAGACCAGCCTAGACATCATAGT
GAGACCACATCTCTACAAAAAAAAAAAAAAAAAAAATTTAATTAGCTGGGTATAGTGGCAC
ACATCTATATTCCCAGCAACTCAGGAAGCTGAAGTAGGAGGATTGCTTGAGCCCAGGAGT
TCAACGTTGCAGTGAACCAAGATCACGCCACTGCACTCCAGCCTGGGCAACAATAATAAT

AACACATTACTACTAACTCCCAACGTTTACACAGTGATTTTCCAGTGCCTTTCATAAACA
TCACCTTAGTTGATATCCATTTCACAGAGGAGAAAATGGAGGCACAGGGAAGAGAAGTGG
TATTTCCCAAGTCTCCCAGGTAGTGGTCTCTCTCCCCAGAGCTTGTGTATCAGGTCAGCT
TACCAGGAATCCTCCCAATCCATGCACTTCACACCCATGCCCTTTGTGATGCACAGAGAG
AATAGACCCATTT**GGAAA**AGGTGCATCTGACTCCAGATACAGAGAAGGGCCTCTGAGACC
CCACCTGAGGGCTCAGTCTTGGACCCCATTCCTAGCTCTCCTGAAATACAGCCCATCTCC
AGATCCCACATGGGGCCAAAGCTGGCTGTTCCACCATAGTTCAGGGCCAGGCGAGCACCA
GTTTGGCCAGGAGAGCCCCAAATAAGTCCCTCGCTTCTGAGAAACCAGGCCATCTGTGCC
TCAGCACTCCCAGAACATCCTTCCCTTCCTCCAGCATATGCATTGGTGATTATTTTTCTG
ATCACGTGTTCACTCT**GGAAA**TAAAAGAGGAAAGTGCTAGCTGGGCTTAATGAGTGCACA
CACCGGCTATTGCATCTGATAATATGCAATGCAAATAAAGGCCCCTTTGAGCCTTCTAAA
ACCAACTATTGAAGTGGAGTCCCCTGGTGCCCCTGAGCCCAGTCAGTAAAATTATTTTAG
AAAATAAAAATAATTGTTGTTATTCATTCCTGCTTACTCTAAAAAGCTTTTTTCTCTTGT
GTTTTTTAATCCAGTAGAAAGAATTTTACAGTAGAGACACAGGGGGTCTTTCACTTTAAA
GCCCAGGATGGGCAACTGATCCTTTGCCATGGGAGGCCATGGGGACAGCTCTGCACTTAC
CCTTGAACAGACAGTGCTGGGCGAGGTTCGGGGAAGCCACTTCTCCCCTCTGAGTCACCC
AGAAAGTGGGCACAGTGACAGTGATGAACTAATCCTGCAGCCATGGGGCTATTCCGAGGA
TGCTCCTAAATTCAGGCTGCCTCTGAACTTCCCTTGGCCTCAGTTTCCCCATCTATAAAA
TGAGAGTGATGAAAATCCCTCCTGCCCCACCTTGCAGGGATACTGGGAAGACTGACAAGA
GATGACACTTGCAAAAGCCCTTTGAAACTGCAAAAGGCTGAACCGTGCAAGTGATTATGA
TGAAGGTAACTGGGACACTCTGGGAGGTGAATGTCTCATCCTGTTTTATGAAAGTGGAAG
CTTGATGAGATTGGGAGATAGAAAGCCAGCAGCTAGTTGGTGGTGAAATGAAGACTTCAG
GAACCCTGTCGCAGCAGTGCCACCTCAACTTCCCTCCCCTGGGCTCAGAGGGGCTGAGGG
GTGTCCATGGTCTCCCTGACGGCAGCTTCTGGAGAAGCAATGGCAGGACGGAAGTAGGCA
CGCACTTTGGGGCCAGCTCTCCATTTACCCATTGAGACCCAAGCAGCTCATCTCCCTCTC
TGAGCCTCAGTTTCTCATCCATAAATGGGTATAATACAAGCACACCCTTCATAGGTGAGG
CTGTGGCGAGGATTAGATGGAATCTCAGAGCTTATGTGTGTGCCCAGCACATGGTGGTGG
ACTGTCCTGGAATGTGAGCTGTGAGAATCACCATTGCAGCCATCATCCAAGGGTGGCCAA
CCTTCTCAGACCCCTCCCAAGGGGGCGAGGCCTGTTGGCAGAGCAGGCAGAGCTCTGGGT
GAGCGTGCAGGCCCAGTAGATGGGAGGGTGCTGGCGAAGGCAGGTGTGCTAGGCAAGAGA
CAGAGGCCTCCATTGCCCACATTGCACACCCAGCCCACAAGCTCCTGGCCAAGGCTGGAG
TGGGGGCCTCCCAGGCAGCTCTGATGAGTCGGTGGGGGGCAGCCAGATGCAGCACCAGGC
CCTGGTATGGAAGGGGATGCCCACGGACACTACTTAGCCTGCTGCTGTAGCCTGAGCCAT
ACCCCTACCAAGGGGTCAGCACCCCCATTGGGGACCAGAGCACTTCATCAAGAACCCGGC
CACACTTGAGGTCCCTCGTATACCACCATGGGGACCCCTGATCACTCTTTGTTCTGGTCA
CTCTCAGGGGCATTGGGCAAGGGGTCATCCTTGGGTGGCTTCACACTCTTCAGAGTGTGA
AGGAGAAAGATCATCCCTCTTGAAGGCCAAAACTAAGGAACACATTGACCTCAGTGCCTT
GTCCAAACTCCTCTCCATTCTAGAAACACTCTCTCCTCTCTCTCTGTCTCTCTTTCTCTC
TCTCTCCCCCTCTCCCCTCTCTCTACTGCATGTTTTCTTTTGTG**GGAAA**ATGAAAAGGAC
GTAACTGAACAAGCTTCCCTTTGCCTTCTCTCAACCCAGAAAACACCAAATCGGTCACAA
AAAGCTTGGTGTGGGGGCCTGAGTGCTTCTCTGCTGCCTCCTCCAGCAGACTCTAATCCC
CTGCACCCTCTGATACTTCCCTTTGATCTGTAACACGCTCAAGGCCAAAAGTATTCCAGC
AGAAACCTCCCTCCTGAGTCCCCTGGAGAATCAAGAGTTGCCCGGCTGGCAGGCCCCACA
GCTAAGGTGCGGCCAGGCCTGCTCCTGCTTCAGGGCCCTCCCCAACCCGTATCCCCACAC
AGAGGGATGCAGTCAGCACGGCCCGCCCTTCATCTGTGACATTAATGTCTGTGGCCTGTT
GCGTCTTCGTGATGACCGACCATGTGTTGTCTGTTCTCTGTTGTCTGTTAAAATTCTTTG
TCGTGTAR**GTAAAATGCAGGATGGCAATGTGGACGCCAGCCAGAGCAAAG**GTAACCMTTT
TGTTTTAACCGGCGTTTGTCGTCCCTGAGGCCCCTCCCCACCGGCTCTGACAGTCACACC
AGAGACAGAGACTGGCAAGGGCCAGAGGCAGACACATCCACTCCTACAGGTGTCTGACGA
CCTTGGGGGTGTAGCTGTGATTTGCATGGTGCCAGGCCAAATAAAGAGGTGTCCACCGGT
GAGGAGGGGGATACCCATGGCCAGCATCTCCCTAGCCTTTGAACTCTGCGCCCCATTCA

GCTGAAGACTTTGAGATCCTTAGAGGTTGAGGGGTAGGGAGTTTGGCTCAGTCTCTGTCC
AAAAGGGTCACGGGCCCTCCAGTCCATCCTGCATGAAGAGGCCACGCCAACTGGGATAGT
GACTTGGCTCCCATGGCTGCCCTCTTTTACGTTCTAAGCCTCCTTTTTTGTCCCTCCTGT
CGAGTGGCTACTTAATATCCCACAACAGGTCCCAAGGACCAGCTGGGTAGAGCCCAAGCC

SEQUENCE BEFORE SPLICED EXONS: 172 bp, 55 bp

Intron 22 – 23

TCAAAAAAAAAAAAAAAAAAAAAAAAAGACATTGTCAAAGACTGGGCTGAGGAACAGGTGA
GGTGGGTGAAGGAGTAGATTGATCACATGCCTGGGTGAGCGGGTAGGAGGAGGGAGGTAG
AGGAGCGGGTGTCAGGGAGAGAAGGCCACTGGGAAGGACAGCCCCACCGTCCTGACAGAC
AGGGTCCCTTGCAG**GTCATCGCCACCATCCCGACCAGCAGACTCAAGTTCCTCAAGGAGG**
CAGGCAGGCTCACACAGAAGGAGGAGATCCCGGAGGAGGAGCTCAACGAGGACGTGGAGG
AGATCGACCACGCGGAGCGGGAGCTGCGGCGGGGCCAGATCCTGTGGTTCCGAGGCCTGA
ATCGGATCCAGACACAGGTAYAGGAGGCTCTTGGGGGCTGGGCCCAGGCCTGGCCGGGGC
GGGCCAGGGGATGGAGGTGAGTGTGCTTGTGCTGCCCCCCAGGGCCCAGCTCTGGTGCTG
AGGGAGGGTGGCTACAGAGGGGTCCCGGCCAGCTCTCACCAGCCACTTGCACACCCACTC
CTGTGGCTGCTAAGGAACAGCACGGCCTCCTCAGAGAGGTCCCATTGTGCCCTCTCAGGG
CAGCACGGGGAAGTGGTAGGGGTCACACCCACCATGGTCTCCAGGGGGATTTCCCTGAGA
TGTCCACAGTCAAGGCACCAGATGTGGCAGAGCCAGGATGCAAGTTGGGTCTTTCTGCCT
CCAAGTCCAGTGCTCTTTCCACTAGCCTTGAAGACTCAATGACCAGCCTCCTCCCATTCT

PMCA2: 16 NFAT motifs

Splice region in PMCA3 gene (ID: ENSG00000067842)

Legend:
GGCGTGTGGGTGTGG – exon
TTTCCC – NFAT binding motif
GGGAAA – NFAT binding motif
TATAAA – TATA box
alternative splicing region
R **– Altered nucleotides recognized during splicing**

SEQUENCE BEFORE SPLICED EXON 42 bp

Intron 2 – 3
AAGGCTCCTGTCTAGAGGGAGAGCCAGGCGCTGGCCAGACGGCCCCAGAGGGCTGCTGCC
ACCCCAGGAGCAGTCAGTCAGGGCCACATAGGAGGCCTGGGACACACAAGGCCGACCCGT
CCCATCCCATCCTCCCCTTCTGTGCTTCCGGGGCACCATGCAG**GCCTGGCGGACAACACC**
AATGACCTGGAGAAGCGCAGGCAGATCTACGGGCAGAACTTCATCCCCCCAAAGCAACCC
AAGACCTTCCTGCAGCTGGTGTGGGAGGCCCTGCAGGACGTGACCCTCATCATCCTGGAG
GTGGCTGCCATCGTCTCTCTGGGCCTCTCGTTCTATGCGCCGCCAGGAGAGGAGAGTGAA
GGTAARSCCCGGGGGCCTGGGCTGGAAGGAGGAAGAGGAATGGGGCTTGAGATGAGGGAC
GGTCACAGGATGGTGATGTCTCCTCCACTGCTTCCCTTCTGTCCTCCRCACAG**CCTGTGG**
GAATGTGTCGGGAGGCGCAGAAGATGAGGGCGAGGCCGAAGCTGGCTGGATCGAGGGGGC
TGCCATCCTGCTGTCCGTCATCTGTGTGGTGCTGGTCACGGCCTTCAATGACTGGAGCAA
GGAGAAGCAGTTCCGAGGCCTGCAGAGCCGAATTGAGCAGGAGCAGAAGTTCACGGTCAT
CCGGAACGGGCAGCTCCTCCAGGTCCCCGTGGCTGCGCTGGTGGTGGGGGACATTGCCCA
GGTCAAGTAYGGTGAGTGCCCTGGTCTTCACCCACCCTGTCAAGGAAGCTCGGCTCCTGC
TTCTGGGGAGGGTGAGCCAGAGGAGACCCCCAGCACAGGTTCTCCCCGGTGGCCTGCGTG
TCGTCACCTGCCTTAGGAGGCGGGAGGTGGCGGGGGTGGGGGAGGTGGGGGAACACGGGG

Intron 3 – 4
CAGGGCAGAGTGGTTTCAGACACAGCAGGCCCCCGAAGAATCAACTGTCCCCACAGCCTC
CACGGCGGGCTTTCCCCCCTACAAAGGATGCAAATGTCCTCGTTCCCCAGAAGGAGGAGG
GCTCCTGAGTCCCTTTCCCAGGCGGCCCATGGCACCTGACGGGACCTCCCCTGGGGCAGC
CGGGAGGAGGCGGTGGGGAGAAAGGCCTCTGCTTCCCGGTGCTYTAG**GCGACCTGCTGCC**
AGCCGACGGCGTGCTCATCCAGGCCAATGACCTCAAGATCGACGAGAGCTCCCTGACGGG
CGAGTCTGACCACGTGCGCAAGTCAGCTGACAAAGATCCCATGCTGCTCTCAGGTGAGGG
CCACCCTCCGGGCCAGCCTGGCACCAAGGGGCGTCAGCAGCCGTGTGGCCCAGCATCCTG
TCCAGGCTGCCTGCTGGGCTCAGCCTCCACCTCCACCAGGGCCTTCCAGCATAACCTTCA
GGTTGGGAATCTGAAGACTCCGCCCGTCTTGAGGCCTGATGGTGGCAGCTCCAGCCACAG

Intron 6 – 7
GTGCTGGGAGGTGGTGACGGGGTGAGCCTGGAGAGGACGGGGCATGAAGAAGGCCACCAG
CAATCAGGCCCCTCCTCCCCACCCCCTCTGCCACGCGGCCCCCAGGCTGGTGTCCTCAAG
CCTTCGTGTCTGTCATCCCTCTTCCATTGTAG**GCAAGCAGCAGGATGGGGCCATGGAGAG**
TAGCCAGACCAAAGGTAACGRGCGCCGCTGCTTGGGCACAACAAGCTTGGAGCCCTGGGG
GTGGGGCTGGTGGAGGGCACAGTGTTGGGGGAGGTTCACCTGCCTCGGTGG**CAGG**AGCAA
CACTTGGAGAC**CAGG**TGAGTGGGCTGGCTGCACAGTCCCGTCCCAGTGCAGAGGGAGGAG

SEQUENCE BEFORE SPLICED EXONS: 154 bp, 68 bp, and 88 bp

Intron 19 – 20

GATCTCTGTGCCGGCCACAGGGGCTGCTGCAGCTGCTGCAGATGCCAGCCTTCTTGCTGG
CCCCAGCATTAGTGTCCTCGGGGACAGCCCACCCAGTTCCTGTTTCATTTTCAGAATTCT
CCTTTCCCTCTGCTACTGCACCTCACCTCCGAATCACACTTGAACCTGGTTGCTCCATGG
AGTCCCTTCACTTCCCGAAACCTGGACTCTAACCACAGCCCAGAGGGGTGCCAGGCCCTC
TTCTTGCTCTTCAAGCTCCCAGGCCACTGGCCTTCTGGTCCCCCCAGGAATGTGGCACCA
GCCTGTAGCTCGGCCTCTCAGGGCTACTGGGTTGTCCTTTTCATTTTTAACCTTGTTCAG
TTACAAAGATAAAACATGTTCAAGAGAAAAACGAAAAGAAGGGAATCTCTGTGAACCAGT
CCTCAAGTCCCCTCCTGGCCATGTTCGACAAATACACTGGCATAGACACACAAGACAGAC
GAGACGTCTTGCTCTAGAACTCCAGATGCCCATCATCCCTTTCTGTGGCTTCCCCTGCGT
CCTTCTAGACTGTTCCCCCCACTTCCCAGTGGCCTACAATATGCGAAGCCATGCCCTCTC
TTTACTCCTGCCAGGCTCCTTATCCAGTCACCCTTTCCAGCTGTCACCCTCCTTGTCACC
TCCCACCCTCTCTAGCCCCCAATGCACAGGTCTTCTCTAGGCTGGTGTTCCTCCAGGAGA
TGCCCTCTCCAGGAGAGACTAGGCCAGAGCTCCAGCTGTCCTCTCCCAGGGGAGTTGCGC
AAACAGCGCCTGTGTCTCCCAGCAACGGTGTGGAGCGACATGCAGGGAGTATTGTCAACC
AGGGCAGCTCACCCAGGCCCCGGTGTCCAGGGCTTTTCTTGCGGTGGTTCACAGAGCCAT
GCTGTGGTCACGTGGCTGGCCTCCATCTCCAGCCCTCTGGAGGTCCAGCTGTTAGTGTAG
CCTACGGGGCACGGTCCCCAGCCCCCTGCCCCACTGAGCCATGCTGCTCTCAAGCAGGAT
GTTCCAGGGGCTTAGCGATCCCTCCCAGGAGCCCTTGCTGCACCGCGTTCCACTCGGGCC
ACTCCCTGGGCCCTTTGGCTGAATGGAGCTCCATGGAGGATCCGCTTATCTCTGTCTTCA
TCAGCTCTGACTGCCATAGCAAAATACCATAGACTGGGAGGCTTAGACAACAGACACTTA
CTTTCTCGAAGTCCCGGAGGCTGGCCCAGAGCTCCACGTACTCCTGAAAATGCATCTCAG
CCCCCCGGGGGAACACTACGCCAGTTCTCCAAGGGCTGCCAGTTCCCGGGGGGACTGCTC
TAGCTTTACTCCCTTTGCTTAGTCACAAAAACAAATGACATCCTTTGGAAGAAGGAGGCC
GGCGTCTCCCTCTGGAGAGGGACCTTACACCAGGCAGATGCTGCTCAGCCTGGCTCTCAC
GGCCACTTCCGTGTGGCTCCCCCAGGT**MATTGCCACCATCCCCACCAGCCAGCTCAAGTG**
CCTGAAGGAAGCCGGGCACGGGCCCGGGAAGGACGAGATGACCGACGAGGAGCTGGCCGA
AGGCGAGGAAGAGATCGACCATGCCGAGCGGGAGCTCCGCAGGGGCCAGATCCTCTGGTT
CCGGGGCCTGAACCGGATTCAGACGCAGGTAAGCCCCGACTCGCTCTCGCCCTGGCACTT
CCACCCCAAGAGGGTAGAGGCAAGCTGGCCAGCAGCAGCCGAGAGCCGGCCGTCTGCACC
CGACACCCACAGGGAAACCCTTTGGTCTTGCTGCCAAGGTCACAAAAGCAGCTGCCAGTG
TCCCCTTTGCCATTCCGGGCTGTGCCCGCTGATGGCTGTGGCACTGAGATCGCCTCTCCC
TGACAGCTCCTTCAGAGCCCAGGCATCCCCTGTAGCTAAAGGTGTGTGTGGGTGACTAAT
GGCTCTCTCAGAGGCACAGGCCAGCACCGTCCTCATCACTGCTGGGGCTCCCTTCACTTG
GTGGGAACGGCCCCAGGTCATAGGCAAGTGACAGTATCGGGAAGAGAGGCCCATTTCAAA
CAGCCACCAGCGAGCTCAAGGTTCACTGGTTGACACTGTAAATGCGATGGGGAGACTTCA
TTTGGGAAGTGGACTCTGGCCACCGAGTTGGTCCTTTCTGGTCATCCAAATCACCAAGGG
TTAGGGCCGGCCAAGACCAAGGGCGTCAGCTGTGTTCCTCTCTGAAGTCAGATGGCCACC
ACTGCCACCAGTATCAGGCGCTTGCCAGAGCTACCATGCTCCCTTGTTCCCATCTCCTCC
CGGCCAACAGTTTTGCCAGAATGCTCCAGACTCGCTGTGTCTCCACACCCCTCCCTGGAA
CTCTCTTGTGATCCCACTACAGCCTGGCCTCTGCCCACCACTCCCCAGAAACGGCGCTCG
CCTAGGCCTGCGAGGCTCCCTTCCGGCTCGTGGGGTCGGGCTGTCCCTGCATCGTTCCTG
CCTGCTGTGGGCACTGCCTGGAGACTTGCTGTCCTCACCCGCCTGGCACCCCCACCTCCT
GCTCAGCCCTCGCCCCACCCTGCTGTGGCCCTTGAGTGCTGAAAATCCTGGCCGGGGAGG

Intron 20 – 21

AAGAAAGGGCCCATGGGGACCCCACCTGCGGCCTGGCCCCTCTCCCACCGCAGCCCCTGC
AGCCTCTCCTTCATGGCCTGCCCCTCCCCCAGCAGCCTCCAGAGGGCCACCCAGCATGCT
TCACACTCCACCTCCTTCTGCAAGGCCTGGGCCACCATCTCTCAGGTCACAGCCATCACT
CCCTGCCCCTCCAGGCCCTGTCCAGGATCCCCAGTTGCAATGCCCCTGACATGGAGCGAC
TGACCCTCCTCCCTGAGGTCCTGCCAGAGTAAGGACACCACTGCACACTTGGCTGCGGAG

CCAGGGCCCGCCTGAGCCACCAGCCCCAGCTCAAGCGGGAATAGGCGGGGCCTGAGGCCA
CACCCCTTCCCAGGGTCAGTCACACATCACCAAGGGCCTGTCCCAAAGAATCATCCATGC
AACTTGCACAAAGCTGTTCTTGGTGCCCTGGGTCCTGTGTTCCCTCCCAGTGGTCTCAAG
GGCCGAGTCTCAAGGGACCCAGCCTCAGGGCAACCCCACTCCCTGGGCCTCAGCCTCCTT
TGCTTGCCTTGGCTCAGCTCAGCCCCTCCAACACCACCCTCTGCAGTGGGACTGGTGGAG
CCTCTCTCCATTGTCCTAAGGATGAAGTCTACGCTCCTGACCATGGCCTTCAAGCCCCTG
CCTCTTGGTGTCACCCCAACCTTCCTGCACACCTGCACCCCTGCACCCCCGGCTGCCTTT
CCAATCATGCCCTCTGCCCGCCTGTGCCACTGCACCTCCCCAACTCCCGCTGCGTCCCCT
GGGCCCTCATGCTTACAAAGCCCTCTTTGACTCCTCTCACTCTCACCACGGGCTCCTTGC
AAAACTTTCCAGAGCTACAGAGTCCCCCAGAGACCACGATCACGCAGGGCTAGGTCAAGG
GGCAACAAAGAACTCCACATTTCAAGAGCCCCCCAGGGGTCCAAGGGGCCTCTCCAGCTG
AGAACCGGGAGGCGAGGCTGGCATCCTAGGCACCGAACACAGCGTAGCCTGGGAAGGATG
TGTTAGGTCTGGACTCTGAACACTGTCACCTATGCCCTGGCCTGGCCAGAACACAGAGAG
CCCCGGTGAGGAAGGGGAGCAGGCTTTGTCGCCAGATGGCCTGCGTCTCCCCTCCCACGT
GCAGGGGCTACTGCCTCAAGCACAATGCTCGGACAGGCCAGGGGTGCAGCACAGATAGTC
TGTTCTCGTTCCCTGCAATAAATATTTCTGCGCTAAATCTTAAACACATCGAATGTTAGA
AGCAGCATTCACATCTCCCCCAGTGCCCTCCTAGCGGCCCAACTGTGCCTTCTACGAGAC
CATGCCTATGGGCCCCCCAGCTGCCCCCAGATGGGCTCTTATCTATCGTACCCCTGTTGT
GCTACAGAGCCCAGAGCTGCCCAAGCCTGGGGTGGGGGGTGAGGACAGCAGGACCTTGCC
CCTCGGCGGCCCACTTCACTCCTGCCGCCGCAGCCGAAGGCGACGTTAGGTTTTCCAGCA
GCTGAGACAACAGCCCTGCCCTGACCTGAAGCATCAGTGGTGTCAGTGGTACCCCGGCCA
CCTGTGTGTCTCGTGTCCTCTGTATGTGGGGGCCGCTGCAGCCTGGCTAGTAGAAGCGAG
TCCCATCGGTCAGAGTCTTAGAGTTCCAGGCTGTTCCAATTGTCGGTGCTCACAGAGACA
CGGGCAGCTGAGTCCCCTGTTTTGCGTATGAGGAAACAGGGTGCACTCTTTCCCCTTGGC
ACCAGCCGATCCGACTCTTCCACGGGCTCCTGACCCCCATGGCTTTTCGCTTTGACTTTG
TCACATCCAACTCACCATGTTTCTCAGGAATGCTGAACCAGTCGGTTCTTTTAGCCAGCT
ATGTAAGAGCATCTGCTCTCTTTGATTCGAAACACCTGCTTGAAAAATGGAGCTAAAGCT
TGGGAGAAGCCCGTCAGGTTGTACGTATGCCCCAAGCCAAGAAAAACATTTCCTAGGCAG
CTGGCCCCAGGTTTCTGCTTTTTGGGAACCACAAAATCGCTCCCCAGATTTTGGAGGCCG
GCACAAGCTTTTCTGTTGCTAAGTGAAGGCATTTTGTAACCTGCTCGCACCATGGTTCCA
TACTAACATATCAACGCACCAGATGGACAGGGTGTCATTCCAGAATAAAAGAGTCCTTCA
AACCCAGAACATGTAGCTTTCTGAAGCGCTCATGACTCGGTCTGTCTTTTTTGCATCCCA
TTATAATGCAAAGAGAAGCTTCGTCAGCAACTGGGCCTTGCTCCCGGCTCAGCTCTTGGG
CTTCCAGGTGTGGGGTGGTGGCGACTGTGGTATTGTCACTGATGTGGCTCTCCCACTGGG
ACACCATCTGTAGCCAGGCCAGACCAATGCAAATCTGAGGCAGGAAAGGCATCTTTCCTA
TCCCTCATCACTTTTGCCGCAGCGAGAGTCCTCTCCTGAATCTAGACAGGAGACCCCCCA
CCCCGCAAGCCACCGCGACGCCATCACATCAACCATCCTCCTTCCGTGCGCTGACACACT
CAGTATGCATTCCTCACGGTCGCAGCAAGTGCCATGAGGTTCCCCTTGTGACAAGCAAGC
GAGTGGGATGTGCTGGGCGGAAGGGGACTGGGCAGGCTGAGAAGAAGGAGGAGTCCTTTC
CACAGCGCGTCAAAGGCTGAAGTGGCGTACATCCTCATTTTGGCCGGTGCAGGGGGAGTC
TGAACACTCAAGCTGCCTTCTTCCACGAAGCCCTGGCCCGTTTCAAGACACCCAGGTGAA
CCTGGTGGCCACCACATAGCACTGCAGGGCTCGGGCTGGAGCAGGTAGCTTTGGGCTCCA
GATGCGCACACTGGCTTTGCTGTTCACATCATCTCCAGTGAGACGGACATGAGTCCCCAT
CCCAGCCAAGGGCAGGTCAGGAAATCCAAAGACACTGGGTCAAGGCCCTCCTGCAGGCCT
CAGTTTCCCCAGCCTTGCTTCCCCTTCAATTTGCAGGAAGCTGTAAGAGTGGGGACAAAG
CGAAGCCACGGCTGCCAGTCGGTGGGAAAACCCCCTCCTCCGCTAGGTCCATTCTTCTGC
TGAAGTTTCGAGGGGGCGCCCCCTGCCCAGTCACTCAGATGAGCCACAGAAGCTGGCGGA
GACGGGCAGGCGGATGGAGCATGGGGCATAAGTCCTCCGGGTTTAGAGGAAGCAGGGCGG
GTTCTAATAGGAGCGTGTCATGTCCCTCAGGTGTGTTGGAGAGGAAAAACAAGGTTGAGA
ACTATGAAGTACTTCCTGATAAGTGGCTTGCAAGGGGCATTTCTGACATGTTTGCAGTGT
GCCCTGACTTGTGACCCCCTTCCCTTCCCAATCTCATGACCACTAACCATCTGCTCAGCA

AACCCTCTCTCGGTGGGTGGCACAGAGGCTGGCCTCAGTCAGGGCCCTGTCCAAACATCG
CCAGCTCCCAGTGAGCGGGGTGACCCGATGAGGCTTTGCTGTCACCCTATGTGCCCAGTG
GTGGCCCCTATCCTCGCCCAGCCCTCCCCTCCGTGATAGCCTGGACAACAGTGCAAGCCA
AACTTTATCTCATTTTCTCTCCCACAG**ATGGAGGTAGTGAGTACCTTCAAGAGAAGCGGT**
TCAGTTCAGGGTGCTGTGCGCCGGCGGTCTTCGGTCCTCAGCCAGCTTCATGAYGTAACC
AATCTTTCTACCCCTACTCACGCAATTCTCTCTGCTGCCAATCCTACCAGTGCTGCTGGG
AGTGAGTCYTGACTTCCCTTTTCTCTCATAAATGGCATCTCTGGGGAAGAATAAGCCTCC
CCAGCTCGGCCTTCTCTCTTTTGCAGTCTTGCATTCCGCTCACCGTGGCTTTTCTGTTCT
TTCTTTACCGCCCTGTCCAGTCCTTCCCGCCCAGACGGTGTCTCTCGAAAGCCATATTCT
TTTATGAAACGTATTCAACCCATGGCACTTCCGGACCTCCCGTTCTTTGCTTTGTTTGGT
TTTTCGGTTTGTGTTTAGTGCTCCTTGTGCCATTGGTTCTCTTTTCTTGAACTTGTCTGC
CTTTGCCGATGGTTCTTCATGAACTTGGGCCGCTTCTGGGGGTGTCTGGTGTCAGGCTAG
GGCCTTGCTCAGTGTTCAGTGGTGCAGCATCAGGGCAGCACGCGGGCTCAGGGCCGCCTT
CCCGGGGCTGGAGGGGTGGCGGGTGCCTTTCCCACACTGTTTGCCACAGAAGAATCTAGC
CCACGTACCCAGGAACTGGAGCGGCGCTTCCAGCATCCTCCTTGAAGTGGTTTCCTAAGG
ATGCCGCTGTCACCTGTCCCCAGGTGGGCTCAGTGCTGACCAATCACTACTGCACAGGTG
GTGCCTGCCCTGTAGGCCTTGCCCCAAGCCACCGGGTGTTAGAGGTTTCCCCAGAACTGG
CACTCAGAGGCCTCTTCAAGACGTTGCCAGGAAAATTTCCCACCATGTTGGTCACAGTTT
AAAGAGAACCCAGGGGCTGTCCTAAGGAGGTAGAAACTTCCAGAATTGCTCACATCTTTT

PMCA3: 21 NFAT motifs

Splice region in PMCA4 gene (ID: ENSG00000058668)

Legend:
GGCGTGTGGGTGTGG – exon
TTTCCC – NFAT binding motif
GGGAAA – NFAT binding motif
TATAAA – TATA box
alternative splicing region
R **– Altered nucleotides recognized during splicing**

SEQUENCE BEFORE SPLICED EXON 36 bp

Intron 1 – 2
GTAGGATATTTGTATATTTGCTTTTTTTCAATCATTCAATTTTATGAACATGGGGTCTAA
GCAGATTTCAAAACAAAGACCATT**TATAAA**ACTGGACCATTTTAATGTTTACAATGTAAT
AAATCTCTTGATTGCAGACATATATGGCTGTTTGGTAGAAACATTACAGTAATGGCAGTT
TTTTCAGTTGGTGTGTAGTCCCCAATAATTATATATGAAATTGCTGTCAAACCAGTAAGA
CTGCATTTATGCAGCCATCATTTT**CAGG**ACTGTTGGTAACCTGAGCATATTTTCTCCAAA
TAACTTTGCCTCCTTGTGTCACAAGGCCCAATTCGCTCACATTTACCTCAATGACAATAT
CTTTGGTAATAACACCCACAGTTGTATACGGTGGGATGAGGGATTCTTCTTTACACCAA
GTATTGGTGGGCAAAAGGTGGCTTTCAGTTCAAGATGTAATACATGGGCTTTCTTGAAAT
GCATATTGGCCTGATCAGTCTTTCTTATTTAGGTGGTTTTCTTGTAAAGCCATCTCCGAC
AAAGCAGAGTTTAGTAACCACCCTCTTCCATGCCTTCTTCTTTCTCTTTCCTGTTCAAAC
AACTTTTAATACGTCTGTTTCTCCCTGGGCATGTACTTTAGTCAGAGGGACTTCCCGTTT
TCCCACCTTCTCTTTTCATTTCTGTTTAATCATATTGGAAAGTACTTTGGCTCGAGATTG
TCCCTCTCTGTCCAGCAGATAAG**CAGG**TACTGCTCCCTGTGGAGTCTTTTCATCCTTCTT
TTGTTTGGTGTTTCTCTTTTCATGCATCTTGATAGTCTTTTTCATTTGTATTTTCTCAGC
ATGACACTGTTTAAGGTAAAGCTTAGCCTTCAGACCAATCATTTGCTTTGCCTTCTTTGA
ACGTTCATGAGCCTCTCGTTCATAAGCCTCTCAGCTTTCCTTCTTTCTTTTCTTCTCATG
GTAATCCAAACGGTATCCATAGTGTTTATGGTGTAATTCAATATATTCGTTCTGTGGCAT
GGTGACAGGCGCAGAACCACCAGGAGTAGCCCCTGAGGTGCGAAAACACTCTCAATTTTA
AGGTCTCAGAGACTCCACGCAGGCCGGGACAGGAAAGGGTCACCCTTTTTTAAAAATATT

Intron 4 – 5
AGTCTCCATGTACTCTCGGCCATTGCTGTCTAGATAGGGCCCATGGGAACAGCCATGAGA
TAGGGTTGAAGGAGTTGGAGGACCCTACCCTCAGCCAGTCTCTTACTATCTCCTTCACTG
TCCCTCC**TTCCC**CTTTGTGYTCTAG**GTGATCTGCTGCCTGCAGATGGAATCCTGATCCAA**
GGGAATGATCTGAAGATTGATGAGAGCTCTCTGACAGGGGAATCTGACCATGTCAAGAAG
TCCCTGGACAAAGACCCCATGTTGCTCTCAGGTAYAGGCCCTGGCTGCCCAAG**TTCCC**AT
TTACCTCCCTGCAAACACCTAGGCCACAGGATCCAGACCCTGTTCTCTCCTCTGACTCTG
TCCCATCTCC**TTCCC**TGGGGTTCAGCTGGGGCAACAGCATTTTGGTGGGTGGAATACAAG
GATATCAGGCAGCCTAAATGAAGTTTTCCATGAAACAAGTGTTATAAGGGCCTTGAAGGG

Intron 5 – 6
GCTTTGCTGGTTGCAGGGGTGGCGGGGGCAGGAGGGAGGGCTTACCCTAAAGCGGTTAGA
CACATTTTCCTCCTTACTCCCAATTTGTCTGGTCTGTTTTAAATGCGCCCAGCTCTTGAC
CTTTTCCTACTGTGCTAAGCACATCATTATCCATGGGGTAGGGAGGAGTATTTTTTCTTC
TAAGTTTGTGTTTCATACCAAATCATGTCTAGGTATTAAAAAACCCATTCC**TTCCC**ATCT
TCTCCTTTCCYGTGTAG**GGACCCATGTCATGGAAGGTTCTGGCCGGATGGTGGTGACAGC**
TGTTGGTGTCAACTCTCAGACTGGAATCATCCTTACTCTCTTGGGGGTCAATGAGGATGA
CGAAGGGGAGAAAAAGAAGAAAGGTAAGGGGCATCTGGAATGAGATTCTCTTTCCTCTCA

TCCCCATGGACAAACAAGGAAGCGAGGGAGCAGATCTGGATAGAAAGCATGGTTGGAAGA
ATCTGCTGCCATGAAGAAAGCAGATATCCAAACTCCTTGCTGAGATAAAGATGTAAATCC
TTGGCTCCAGGGAGCTCTGATGAAAACCAGATCCTGGCTCTAGGCTCCCAAATCAGGTTG

SEQUENCE AFTER SPLICED EXON 175 bp

CTTATCCCCTATAGGCAAGACAACAAATAGAAAAGT**GGAAA**AGAGGCCGGGTGCGGTGGC
TCACGCCTGTAATCCCAGCACTTTGGGAGGCCGAGGCTGGCAGATCATAAAGTCAGGAGT
TCGAGACCAGCCTGGCCAACATAGTGAAACCTTGGCTCTGCCAAAAATACAAAAATTAGC
TGGACGTGGTGGTGGGCGCCTGTAATCCCAGCTACTCTGGAGGCTGAGGCAGGAGAATCG
CTTGAACCCGGAAGGCTGAGGTTGCAGTGAGCTGAGATCGCGCCATTACACACCAGCCTG
GGCAACAAGGCAAGACTCTGTCTCAAAAAAAAAAAAAAAAAAAGAAGAAGAAAAGTGGATT
GCAGACTCACTATGCACCTGACTGTACTTCCAGGCAGGTGCTTTTTCTGTCTGCCAGAGA
AACATTCCAGGGTGCTGTGGCTGCCTCACCTATCCAGGGCGATGCAGCTCCCTGGGGACA
CAGGTGCTTCCTGGGGAGCCCTGAGCACTTCTCCGTGTTGCTGGCCTCACATCCAATTGG
GCAGGCATTGGAGTCCGGGCACTGCCTGGAGCTCACCAGAAGGTGCTTTACAGTGGTCCT
TGGTTTGTCTCTCAGAGGGCTTGGTCCCCTCTGCTGTGAGGCTGGACTGCAGCTGTGGTG
GCAAGTGACCAGATGTCACTGAGCAGCCTGACCATGGTCTAGGTGGTGCTTGGATGCATC
CACTTGGCTTCCTTTGGTCATTGAGTTTCAAGAAGAGAAACTCCAGGAGACGGCTTGGCC

AATGCAGCTTTCAAAACGTCCTAGTTGCCGTCACTCACCCCAGCCCACCCCACTGCTTGA
AAGTTGAGCCATGTGATGTTTCTAGTGCCCCATGATCTCTTCTGATGTGTGTTTGGTTTT
GATGCTGAGCAGTCATGTTTTATAGTCTGGGTTTTGTTTCTCTCCTAAATCCACCATCTC
CTTGTTCTGCCGGCCAATTCTCACCTCTCTATTTTCTCATCCTCCTTTTCC**TTCCC**CTGG
TAYAG**ATCAAAGTGGTCAAAGCGTTCCATAG**TTCCC**TCCACGAAAGCATTCAGAAACCCT**
ACAACCAAAAGTCCATCCACAGCTTCATGACCCACCCTGAATTYGCCATARAGGAGGAGT
TGCCACGAACACCACTCCTGGATGAGGAAGAGGAGGAAA**ATCCTGACAAGGCTTCTAAGT**
TTGGGACTAGGGTGCTCCTGTTGGATGGTGAGGTCACTYCATATGCCAATACAAACAACA

PMCA4: 10 NFAT motifs
