# Supplementary material for: NFAT1 and NFAT3 Cooperate with HDAC4 during Regulation of Alternative Splicing of PMCA Isoforms in PC12 Cells
Source: PLoS One. 2014 Jun 6;9(6):e99118. doi: 10.1371/journal.pone.0099118 (PMC4048221; doi:10.1371/journal.pone.0099118)
Supplement: Table S1 — Antibodies used in this study. (DOCX) [file pone.0099118.s003.docx]

**Table S1. Antibodies used in this study.**

| **protein name** | **host organism** | **predicted gel band size (kDa)** | **dilution** | **supplier** |
| --- | --- | --- | --- | --- |
| ß-actin | mouse | 43 | 1:5000 | Calbiochem |
| PMCA1 | rabbit | 130 (**1b**), 134 (**1a**) | 1:1000 | Abcam |
| PMCA2 | rabbit | 127 (**2b**) 133 (**2a**) | 1:2000 | Affinity Bioreagents |
| PMCA3 | rabbit | 127 (**3a, 3b**) | 1:1000 | Affinity Bioreagents |
| PMCA4 | mouse | 129 (**4b**), 133 (**4a**) | 1:1000 | Abcam |
